# Supplementary material for: Transcriptomic analysis of early stages of intestinal regeneration in Holothuria glaberrima
Source: Sci Rep. 2021 Jan 11;11:346. doi: 10.1038/s41598-020-79436-2 (PMC7801731; doi:10.1038/s41598-020-79436-2)
Supplement: Supplementary file 1 — Supplementary Figures. [file 41598_2020_79436_MOESM1_ESM.pdf]

Transcriptomic Analysis of Early Stages of Intestinal Regeneration in *Holothuria glaberrima*

David J. Quispe-Parra[1], Joshua G. Medina-Feliciano[1], Sebastián Cruz-González[1], Humberto Ortiz-Zuazaga[2], José E. García-Arrarás[1*]

[1]University of Puerto Rico, Biology Department, San Juan, 00925, Puerto Rico.
[2]University of Puerto Rico, Department of Computer Sciences, San Juan, 00925, Puerto Rico.

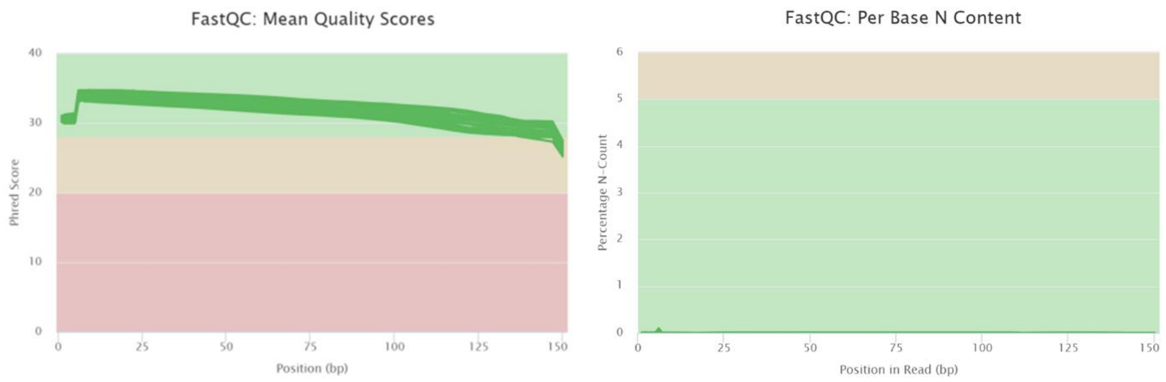

Figure S1. Quality assessment of reads after processing with Trimmomatic. All reads obtained a Phred score of 30 (left) meaning a 99.9% of accuracy and a null per base N content (right).

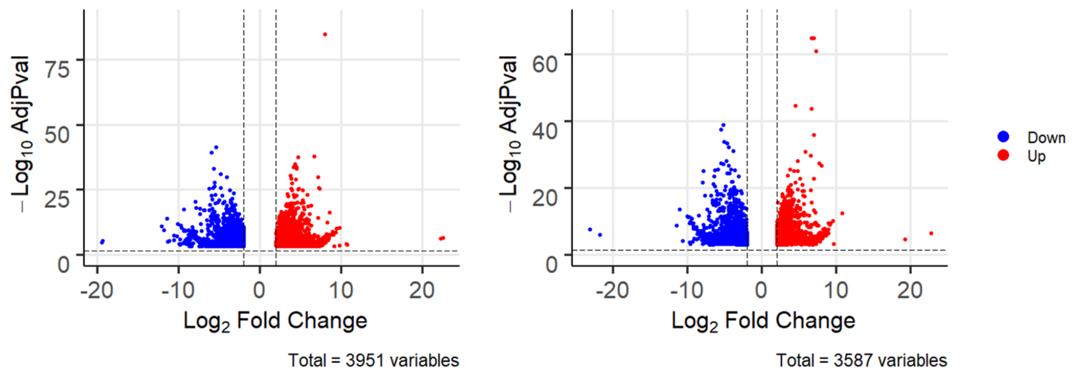

Figure S2. Differential gene expression analysis with adjusted p-value < 0.001.
