# Supplementary material for: Transcriptomic analysis of early stages of intestinal regeneration in Holothuria glaberrima
Source: Sci Rep. 2021 Jan 11;11:346. doi: 10.1038/s41598-020-79436-2 (PMC7801731; doi:10.1038/s41598-020-79436-2)
Supplement: Supplementary file 2 — Supplementary Tables. [file 41598_2020_79436_MOESM2_ESM.pdf]

Transcriptomic Analysis of Early Stages of Intestinal Regeneration in *Holothuria glaberrima*

David J. Quispe-Parra[1], Joshua G. Medina-Feliciano[1], Sebastián Cruz-González[1], Humberto Ortiz-Zuazaga[2], José E. García-Arrarás[1*]

[1]University of Puerto Rico, Biology Department, San Juan, 00925, Puerto Rico.

[2]University of Puerto Rico, Department of Computer Sciences, San Juan, 00925, Puerto Rico.

Table S1. Results of transcriptome assessment with BUSCO

Parameter	BUSCO result
**Core genes queried**	978
**Complete core genes detected**	99.1%
**Complete single copy core genes**	27.2%
**Complete duplicated core genes**	71.9%
**Fragmented core genes detected**	0.4%
**Missing core genes**	0.5%

Table S2. Transcriptome length statistics and composition assessments with gVolante

Parameter	Result
**Number of sequences**	491 436
**Total length (nt)**	408 930 895
**Longest sequence (nt)**	34 610
**Shortest sequence (nt)**	200
**Mean sequence length (nt)**	832
**N50 sequence length (nt)**	1 691

Table S3. RNA-seq data read statistic values

Accession (SRA)	Sample	Quantity of reads		Mapped reads
		Before Filtering	After Filtering	
SRR12564573	NormalA	89 424 588.00	88 112 720.00	94.18%
SRR12564572	NormalB	74 105 862.00	72 983 434.00	93.79%
SRR12564570	NormalC	60 626 094.00	59 767 896.00	91.55%

SRR12564564	Day1A	32 499 910.00	31 721 976.00	90.67%
SRR12564563	Day1B	35 164 514.00	34 237 960.00	91.37%
SRR12564571	Day1C	43 519 280.00	42 179 220.00	91.53%
SRR12564567	Day3A	64 094 536.00	62 995 792.00	90.26%
SRR12564566	Day3B	71 654 462.00	70 158 078.00	91.07%
SRR12564565	Day3C	71 259 560.00	70 418 332.00	90.75%
SRR12564569	Day3D*	107 688 184.00	106 083 898.00	-
SRR12564568	Day3E*	108 372 334.00	106 767 522.00	-

*Samples used for the assembly but not for differential expression analysis

Table S4. Genes and primers used for RT-qPCR

Gene	Primers
**VBP**	F: AGGTCAAAATGTCCGGCCTCTGT<br>R: TCACGCTGGCGGTGTCAAAACT
**FoxA**	F: ACGGCGAAGGTAGCATCCGTTT<br>R: ATGGCAATCCAGCAAGCGCCAA
**Wnt6**	F: CCTGCAATAATCCGGTGAGT<br>R: CGCGAATGTAAATGTCATGG
**Sox4**	F: CGCTCTCCATTTCAGAGGAC<br>R: GTCTTTCTGCCTCCTCAACG
**Tap26**	F: TGCCTGAGCTTTCTGTTCCT<br>R: CCCTCAAAGAATGGTGGAAA
**NADH**	F: CAATGGTTGTTGCTGGAGTCTTT R:<br>CGCAGAAGTAGCCGCGAATAT
**Tenascin-N**	F: CGTTGAAATACCGTCCATCC<br>R: AACGAAGGATACGCTGGAGA

Table S5. Mean Ct values obtained from RT-qPCR

Gene	Stage	Ct Mean	Ct SD
**VBP**	Normal	28.636	1.268
	Day 1	28.263	0.640
	Day 3	27.958	0.738
**FoxA**	Normal	23.611	0.371
	Day 1	27.643	3.124
	Day 3	27.824	3.201
**Wnt6**	Normal	28.607	1.399

	Day 1	25.253	1.965
	Day 3	27.842	3.703
Sox4	Normal	26.038	0.784
	Day 1	25.424	1.143
	Day 3	29.242	1.014
Tap26	Normal	27.634	1.319
	Day 1	26.727	0.483
	Day 3	29.512	1.311
NADH	Normal	18.843	0.271
	Day 1	20.829	0.355
	Day 3	22.583	0.341
Tenascin	Normal	21.025	3.217
	Day 3	30.992	1.519

Table S6. Comparison of expressed transcripts in RNA-seq with previous studies

Gene	RNA-seq		RT-PCR/qPCR	Northern Blot/In situ
	Day 1 (Log2FC)	Day 3 (Log2FC)	Day 3	Days 2-3
**Myc**	2.72 ↑	2.05 ↑	↑	NA
**β-catenin**	0.88	0.46[NS]	-[NS]	NA
**Melanotransferrin**	3.55 ↑	3.7 ↑	↑	NA
**Serum amyloid A protein**	2.39 ↑	3.2 ↑	NA	↑
**WNT9**	2.69 ↑	2.88 ↑	NA	↑
**BMP**	3.53 ↑	3.31 ↑	NA	↑
**survivin**	-0.90[NS]	-0.31[NS]	-[NS]	-

↑: Upregulated; ↓: Downregulated; -: No significant change; NS: Not significant (Pvalue > 0.05)

Table S7. Comparison of expressed transcripts in RNA-seq with microarray study

Gene	RNA-seq	Microarray

	Day 1 (Log2FC)	Day 3 (Log2FC)	Day 3
**Tensc-R**	3.26↑	2.03 NS	↑
**Actin-1**	6.12↑	7.47↑	↑
**Actin-2**	1.71↑	1.56↑	↑
**Collagen alfa-1**	-1.54↓	-0.67 NS	↑
**Laminin alpha1**	-3.67↓	-2.79↓	↑
**Stromelysin-3 (MMP-11)**	-4.16↓	-2.65↓	↑
**Hox9**	1.03↑	-0.83 NS	↑
**Krueppel like**	-1.09↓	0.12 NS	↓
**Gelsolin**	-0.79 NS	-2.69↓	↓

↑: Upregulated; ↓: Downregulated; -: No significant change; NS: Not significant (Pvalue > 0.05)

Table S8. Differentially expressed transcripts between day 3 vs 1 comparison

ID	Description	Log2FC	Adjusted p-value
**Transcript_223195**	15-hydroxyprostaglandin dehydrogenase [NAD(+)]	2.13	3.58E-02
**Transcript_298217**	4-aminobutyrate aminotransferase, mitochondrial	2.98	2.81E-05
**Transcript_334800**	40S ribosomal protein S15	6.73	1.89E-04
**Transcript_254071**	actin, cytoskeletal 3B|actin, cytoskeletal 3	3.00	1.19E-02
**Transcript_432551**	actin, muscle	3.65	5.45E-03
**Transcript_383802**	adenylyltransferase and sulfurtransferase MOCS3-like	3.21	3.38E-02
**Transcript_308851**	alcohol dehydrogenase class-3	2.33	1.59E-02
**Transcript_425811**	calreticulin	2.45	9.78E-08
**Transcript_298293**	collagen alpha-1(XII) chain|tenascin-X	2.08	7.99E-05
**Transcript_196327**	creatine kinase, flagellar	5.16	4.69E-02
**Transcript_380459**	cryptochrome-2	2.03	3.75E-03
**Transcript_226806**	cytochrome P450 3A24	2.25	1.83E-02
**Transcript_262552**	cytochrome P450 3A9	4.03	3.31E-02
**Transcript_175314**	cytochrome P450 4V2	2.00	4.85E-02
**Transcript_172290**	cytosol aminopeptidase	2.21	1.97E-05

**Transcript_184414**	dehydrogenase/reductase SDR family member 7	2.25	1.39E-03
**Transcript_175770**	deleted in malignant brain tumors 1 protein	2.65	2.63E-02
**Transcript_461577**	deoxyribodipyrimidine photo-lyase	2.68	2.66E-03
**Transcript_432963**	DNA replication licensing factor mcm5	2.57	4.81E-07
**Transcript_359454**	dynein light chain LC6, flagellar outer arm	2.35	6.25E-04
**Transcript_347359**	ectonucleotide pyrophosphatase/phosphodiesterase family member 7-like	3.16	4.27E-03
**Transcript_273238**	epidermal growth factor-like protein 7	2.49	1.23E-05
**Transcript_276903**	ERI1 exoribonuclease 2	2.86	3.80E-03
**Transcript_365063**	extracellular transglutaminase	2.62	5.99E-09
**Transcript_350713**	fibrillin-1	2.08	9.82E-03
**Transcript_356914**	ficolin-2-like	3.74	1.04E-02
**Transcript_179574**	ficolin-2-like	3.05	3.76E-02
**Transcript_221519**	formin-J	2.02	1.31E-04
**Transcript_358088**	fucose mutarotase	2.46	1.86E-08
**Transcript_351559**	G patch domain-containing protein 3	4.02	5.91E-03
**Transcript_185057**	GDH/6PGL endoplasmic bifunctional protein|hexose-6-phosphate dehydrogenase (glucose 1-dehydrogenase)	2.07	2.55E-06
**Transcript_402303**	GTP 3',8-cyclase, mitochondrial|cyclic pyranopterin monophosphate synthase, mitochondrial|molybdenum cofactor biosynthesis protein 1	2.32	8.19E-03
**Transcript_438891**	H2.0-like homeobox protein|Homeobox domain-containing protein	3.45	1.75E-11
**Transcript_251717**	hairy/enhancer-of-split related with YRPW motif protein 1	3.17	2.39E-02
**Transcript_456276**	heparan sulfate glucosamine 3-O-sulfotransferase 1	3.31	1.15E-02
**Transcript_438353**	homeobox protein EMX1	3.86	8.19E-03
**Transcript_290553**	isoamyl acetate-hydrolyzing esterase 1 homolog	2.80	5.95E-05
**Transcript_409717**	isochorismatase domain-containing protein 2|isochorismatase domain-containing protein 2, mitochondrial	2.95	4.66E-03
**Transcript_258900**	KRP170	2.69	1.74E-02
**Transcript_189831**	L-gulonolactone oxidase	2.45	6.22E-03
**Transcript_297492**	laminin subunit alpha-2|laminin subunit alpha-1	2.21	1.74E-05
**Transcript_305145**	LOW QUALITY PROTEIN: DNA replication licensing factor mcm2	2.94	6.14E-07
**Transcript_381204**	LOW QUALITY PROTEIN: transcription factor Sox-10	3.54	6.83E-09
**Transcript_285150**	LOW QUALITY PROTEIN: zygotic DNA replication licensing factor mcm6-B	2.29	2.08E-07
**Transcript_294196**	low-density lipoprotein receptor-related protein 1	2.14	2.64E-04
**Transcript_328691**	MAM and LDL-receptor class A domain-containing protein 1	2.14	7.82E-04
**Transcript_257273**	methylsterol monooxygenase 1	3.02	3.79E-03

**Transcript_324570**	microfibril-associated glycoprotein 4|tenascin-N	2.48	3.32E-02
**Transcript_263515**	polycystic kidney disease protein 1-like 2	2.32	1.61E-02
**Transcript_269460**	probable D-lactate dehydrogenase, mitochondrial|lactate dehydrogenase D	3.55	2.01E-02
**Transcript_236563**	protein FAM166B-like	17.42	4.96E-04
**Transcript_437843**	protein lin-52 homolog	2.10	2.16E-03
**Transcript_375637**	protein Wnt-6	2.25	2.75E-03
**Transcript_197666**	putative aminopeptidase W07G4.4	2.06	2.59E-04
**Transcript_311247**	putative hydroxypyruvate isomerase	2.52	5.99E-04
**Transcript_443748**	pyridine nucleotide-disulfide oxidoreductase domain-containing protein 2|pyridine nucleotide-disulphide oxidoreductase domain 2	2.41	1.73E-02
**Transcript_297199**	ryncolin-1-like	5.18	3.37E-02
**Transcript_406209**	sulfite oxidase	3.85	2.99E-07
**Transcript_54760**	testis-specific serine/threonine-protein kinase 4-like	5.30	3.42E-02
**Transcript_251414**	transcription factor Sp5	2.40	4.19E-03
**Transcript_384474**	transmembrane protein KIAA1109	2.08	1.48E-05
**Transcript_266841**	tuftelin	2.47	1.33E-02
**Transcript_368226**	ubiquitin	6.29	1.18E-03
**Transcript_270219**	ubiquitin-40S ribosomal protein S27a	6.47	4.00E-04
**Transcript_437026**	uncharacterized protein K02A2.6-like	3.43	2.72E-05
**Transcript_190019**	uncharacterized protein K02A2.6-like|Reverse transcriptase domain-containing protein	2.32	1.79E-02
**Transcript_291904**	uncharacterized protein LOC105438010|Glycoside hydrolase family 31 domain containing protein	2.14	3.79E-04
**Transcript_283678**	uncharacterized protein LOC583353|neurotrypsin	4.75	9.63E-03
**Transcript_26623**	uncharacterized protein LOC592324	7.59	2.53E-06
**Transcript_442525**	uncharacterized protein LOC753842|Protease inhibitor I35 (TIMP) domain containing protein	2.01	2.72E-04
**Transcript_270528**	uncharacterized protein LOC757055|glyoxylate/hydroxypyruvate reductase A HPR2	2.01	7.71E-05
**Transcript_263687**	valacyclovir hydrolase-like	2.34	1.86E-03
**Transcript_428635**	zygotic DNA replication licensing factor mcm3	2.64	3.93E-04
**Transcript_167684**			
**Transcript_294311**	-	-3.44	2.27E-02
**Transcript_251847**	-	-2.32	7.18E-05
**Transcript_254514**	-	-2.01	2.89E-02
**Transcript_395263**	14-3-3 family protein artA|14-3-3 protein 3	-5.90	2.83E-03
**Transcript_285870**	26S proteasome non-ATPase regulatory subunit 5	-3.03	1.24E-09

**Transcript_42693**	26S proteasome regulatory subunit 6A-B|26S protease regulatory subunit 6A-B|26S protease regulatory subunit 6A-like	-2.16	6.22E-06
**Transcript_48454**	40S ribosomal protein S13	-3.47	4.95E-02
**Transcript_345624**	40S ribosomal protein S16	-3.43	4.17E-02
**Transcript_399286**	40S ribosomal protein S9	-4.03	1.07E-03
**Transcript_382713**	60S ribosomal protein L10a	-2.42	9.80E-03
**Transcript_6160**	60S ribosomal protein L19	-3.63	1.40E-03
**Transcript_1415**	60S ribosomal protein L27a	-3.01	1.48E-02
**Transcript_110850**	60S ribosomal protein L3	-4.01	1.67E-05
**Transcript_274172**	60S ribosomal protein L3	-3.54	3.44E-03
**Transcript_242646**	60S ribosomal protein L31	-3.19	2.25E-02
**Transcript_258108**	60S ribosomal protein L8	-4.33	5.37E-05
**Transcript_438569**	acetylcholine receptor subunit beta|gamma-aminobutyric acid receptor subunit gamma-2	-2.21	8.54E-03
**Transcript_310621**	acid-sensing ion channel 1A	-4.52	9.37E-03
**Transcript_388716**	actin CyI, cytoplasmic	-4.82	1.87E-02
**Transcript_305867**	actin CyI, cytoplasmic	-2.41	1.99E-02
**Transcript_198859**	actin-5C	-2.66	1.48E-02
**Transcript_407345**	actin, muscle	-2.21	2.99E-07
**Transcript_225582**	adenylate cyclase type 3	-2.06	2.98E-03
**Transcript_360808**	ADP-ribosylation factor	-2.02	9.73E-04
**Transcript_197796**	alpha-1 collagen	-3.43	3.20E-11
**Transcript_198232**	alpha-1 collagen	-2.61	2.61E-08
**Transcript_396043**	alpha-1 collagen	-2.18	6.97E-05
**Transcript_345429**	alpha-amylase 4N	-2.34	2.95E-03
**Transcript_410512**	alpha-crystallin B chain	-2.94	1.49E-03
**Transcript_430725**	alpha-crystallin B chain	-2.03	1.93E-05
**Transcript_195199**	angiopoietin-4-like	-2.43	9.40E-03
**Transcript_211686**	arylsulfatase	-4.17	1.71E-03
**Transcript_234345**	barH-like 2 homeobox protein	-3.23	7.50E-05
**Transcript_374682**	bromodomain-containing protein 4-like|dentin sialophosphoprotein	-2.12	6.69E-03
**Transcript_203368**	calcitonin gene-related peptide type 1 receptor	-2.32	5.00E-05
**Transcript_82280**	calcium-activated chloride channel regulator 1|epithelial chloride channel protein	-2.04	4.83E-06
**Transcript_454815**	calmodulin	-3.16	1.16E-02
**Transcript_346536**	calmodulin	-2.13	5.15E-06
**Transcript_60050**	cAMP-dependent protein kinase catalytic subunit 1|catalytic subunit of cAMP-dependent histone kinase	-2.62	7.18E-05
**Transcript_7357**	cardioacceleratory peptide receptor-like	-2.28	2.73E-02
**Transcript_387252**	cathepsin Z	-8.84	1.47E-08
**Transcript_443599**	cholecystokinin receptor type A	-2.23	1.66E-02
**Transcript_198131**	cyclin-dependent kinase 20-like	-2.29	2.97E-05

**Transcript_338163**	cysteine and glycine-rich protein 1|cysteine and glycine-rich protein 2	-2.87	2.40E-16
**Transcript_53888**	cytochrome P450 27C1|25-hydroxyvitamin D-1 alpha hydroxylase, mitochondrial|probable cytochrome P450 49a1	-2.45	2.73E-03
**Transcript_179647**	D(1) dopamine receptor	-2.15	1.15E-02
**Transcript_213703**	deleted in malignant brain tumors 1 protein	-3.53	2.76E-03
**Transcript_321418**	deleted in malignant brain tumors 1 protein	-2.73	2.76E-03
**Transcript_74981**	disintegrin and metalloproteinase domain-containing protein 23-like|disintegrin and metalloproteinase domain-containing protein 12	-2.32	8.72E-03
**Transcript_455894**	DNA-directed RNA polymerases I, II, and III subunit RPABC3	-2.59	9.85E-03
**Transcript_44175**	E-selectin-like	-2.09	6.66E-03
**Transcript_412294**	E3 ubiquitin-protein ligase TRIM56-like	-4.42	3.14E-02
**Transcript_366986**	fibrinogen C domain-containing protein 1	-2.20	3.17E-02
**Transcript_298990**	ficolin-1-like	-4.27	5.65E-06
**Transcript_366365**	ficolin-1-like	-2.21	1.60E-02
**Transcript_259611**	ficolin-2	-2.54	1.29E-02
**Transcript_435128**	formin-J	-2.24	2.03E-05
**Transcript_86447**	G-protein coupled receptor 54	-2.30	3.74E-02
**Transcript_380362**	glyceraldehyde-3-phosphate dehydrogenase	-3.84	1.53E-02
**Transcript_205220**	glycine receptor subunit alpha-4	-2.27	6.61E-12
**Transcript_288548**	glycine-rich cell wall structural protein 1|glycine-rich protein DOT1	-2.14	3.14E-02
**Transcript_305980**	golgin subfamily A member 6-like protein 22	-2.25	1.60E-02
**Transcript_264943**	GTP-binding protein Rit1-like|GTP-binding protein Rit1 pseudogene	-2.07	4.07E-03
**Transcript_281300**	hairy/enhancer-of-split related with YRPW motif protein|uncharacterized protein LOC593175	-2.17	2.97E-02
**Transcript_281299**	heat shock cognate 71 kDa protein|heat shock 70kDa protein 8	-2.96	1.97E-04
**Transcript_300969**	heat shock cognate 71 kDa protein|heat shock 70kDa protein 8	-2.37	8.25E-03
**Transcript_200327**	histamine H2 receptor-like	-4.30	7.87E-04
**Transcript_338151**	histidine triad nucleotide-binding protein 3	-2.68	4.10E-03
**Transcript_161417**	histone H3, embryonic	-3.59	1.41E-02
**Transcript_276623**	histone H4	-4.81	4.60E-02
**Transcript_212508**	histone H4	-3.12	3.75E-02
**Transcript_339518**	homeobox protein Hox-A7	-2.50	3.18E-02
**Transcript_394760**	homeodomain protein	-4.44	2.56E-03
**Transcript_436417**	IgGFc-binding protein|Fc fragment of IgG binding protein|zonadhesin	-2.19	6.67E-03
**Transcript_374429**	ileal sodium/bile acid cotransporter-like	-4.01	8.60E-03
**Transcript_279040**	kelch-like protein 2	-2.18	3.64E-05
**Transcript_219771**	Krueppel-like factor 13	-2.29	2.33E-03

**Transcript_376962**	lactase-phlorizin hydrolase	-2.76	3.76E-02
**Transcript_274293**	lambda-crystallin homolog	-2.16	2.13E-05
**Transcript_429112**	large neutral amino acids transporter small subunit 2	-2.27	1.22E-05
**Transcript_204036**	serine/threonine-protein kinase mos	-2.13	2.72E-04
**Transcript_229121**	serine/threonine-protein kinase mos	-2.02	2.54E-03
**Transcript_257994**	LOW QUALITY PROTEIN: MMP37-like protein, mitochondrial|MMP37-like protein, mitochondrial	-2.04	4.07E-02
**Transcript_391508**	LOW QUALITY PROTEIN: tubulin alpha-1 chain	-2.73	2.20E-08
**Transcript_362387**	LOW QUALITY PROTEIN: tubulin alpha-1 chain	-2.36	6.65E-04
**Transcript_420771**	LOW QUALITY PROTEIN: tubulin alpha-1C chain	-2.16	2.86E-02
**Transcript_364074**	LOW QUALITY PROTEIN: uncharacterized protein LOC588722	-3.12	4.00E-04
**Transcript_339876**	LOW QUALITY PROTEIN: zinc finger protein 708-like	-3.37	1.48E-02
**Transcript_273801**	metabotropic glutamate receptor 8	-2.22	4.80E-05
**Transcript_59717**	methylmalonic aciduria and homocystinuria type C protein homolog	-2.07	3.76E-03
**Transcript_343882**	microfibril-associated glycoprotein 4	-4.38	4.85E-03
**Transcript_216921**	microfibril-associated glycoprotein 4	-2.53	1.48E-04
**Transcript_410262**	microfibril-associated glycoprotein 4-like	-4.02	2.18E-02
**Transcript_179645**	microfibril-associated glycoprotein 4-like	-2.94	3.25E-03
**Transcript_445086**	microfibril-associated glycoprotein 4-like	-2.41	1.99E-03
**Transcript_429455**	microfibril-associated glycoprotein 4-like	-2.38	4.46E-03
**Transcript_220874**	microfibril-associated glycoprotein 4|tenascin-N	-9.00	5.41E-03
**Transcript_362512**	microfibril-associated glycoprotein 4|tenascin-N	-4.39	4.85E-03
**Transcript_335205**	microfibril-associated glycoprotein 4|tenascin-N	-2.89	8.65E-03
**Transcript_367362**	microfibril-associated glycoprotein 4|tenascin-N	-2.64	5.42E-03
**Transcript_343707**	microfibril-associated glycoprotein 4|tenascin-N	-2.25	3.28E-02
**Transcript_365407**	microfibril-associated glycoprotein 4|tenascin-N	-2.22	8.36E-06
**Transcript_343323**	microfibril-associated glycoprotein 4|tenascin-N	-2.11	9.52E-03
**Transcript_264072**	monocarboxylate transporter 12|retinol dehydrogenase 8|Short-chain dehydrogenase/reductase SDR domain containing protein	-2.68	5.62E-03
**Transcript_429832**	muscle-specific protein 20	-2.01	3.32E-04
**Transcript_414296**	NADPH oxidase 5	-2.55	1.02E-03
**Transcript_341092**	neurogenic differentiation factor 4	-2.19	2.56E-02
**Transcript_398720**	neuronal acetylcholine receptor subunit alpha-3	-2.89	3.68E-13
**Transcript_183115**	neurotrophin 5|prepro-neurotrophin	-2.35	3.71E-07

**Transcript_254653**	nucleolar GTP-binding protein 2	-2.17	7.10E-05
**Transcript_420284**	orexin receptor type 2	-2.50	2.06E-04
**Transcript_321671**	oxytocin receptor|gonadotropin-releasing hormone receptor	-3.48	5.51E-04
**Transcript_196171**	paired box protein Pax-2a	-2.30	3.24E-04
**Transcript_335269**	peptidyl-prolyl cis-trans isomerase	-4.41	2.41E-03
**Transcript_418613**	phospholipid scramblase 2|phospholipid scramblase family member 5	-2.09	6.80E-03
**Transcript_391646**	PIN2/TERF1-interacting telomerase inhibitor 1	-2.41	2.09E-04
**Transcript_348719**	popeye domain-containing protein 3	-2.49	8.96E-13
**Transcript_208618**	potassium channel subfamily K member 9	-2.68	4.58E-04
**Transcript_297999**	probable cationic amino acid transporter|putative cationic amino acid transporter|solute carrier family 7 (orphan transporter), member 14	-2.43	2.93E-03
**Transcript_255002**	probable threonine protease PRSS50	-2.87	9.99E-09
**Transcript_217497**	protein giant	-2.12	1.54E-04
**Transcript_303695**	protein PLANT CADMIUM RESISTANCE 3-like	-2.66	6.01E-04
**Transcript_409861**	protein SSUH2 homolog	-2.28	1.53E-02
**Transcript_380758**	protein SSUH2 homolog	-2.22	4.36E-05
**Transcript_315415**	protein Tob1	-2.75	1.53E-02
**Transcript_405702**	putative uncharacterized protein CXorf58	-2.53	9.26E-04
**Transcript_483697**	rab3 GTPase	-2.08	1.81E-03
**Transcript_426847**	ras-related protein ORAB-1	-4.04	8.23E-03
**Transcript_182361**	retinol dehydrogenase 8	-2.15	1.46E-02
**Transcript_269277**	rho GTPase-activating protein 25	-2.73	7.41E-03
**Transcript_367031**	ribosomal protein S14	-3.32	1.75E-02
**Transcript_350598**	ribosome biogenesis protein bop1-B	-2.08	4.29E-03
**Transcript_373223**	RNA 3'-terminal phosphate cyclase-like protein	-2.05	3.54E-03
**Transcript_253908**	RNA polymerase II subunit A C-terminal domain phosphatase SSU72	-2.44	3.37E-05
**Transcript_300509**	ryncolin-1-like	-3.08	4.21E-03
**Transcript_370378**	serine/threonine-protein kinase mos	-2.03	3.42E-02
**Transcript_381580**	serine/threonine-protein kinase NLK|serine/threonine-protein kinase NLK2	-2.27	3.18E-02
**Transcript_285582**	short-chain collagen C4	-2.84	5.23E-04
**Transcript_285813**	splicing factor 3A subunit 2	-2.31	1.66E-04
**Transcript_309701**	sugar phosphate exchanger 3	-2.12	3.13E-02
**Transcript_179855**	sushi, von Willebrand factor type A, EGF and pentraxin domain-containing protein 1	-2.49	3.74E-03
**Transcript_236683**	synaptotagmin-15	-2.28	2.41E-03
**Transcript_275400**	synaptotagmin-17-like	-2.77	1.50E-02
**Transcript_229301**	synaptotagmin-7	-2.73	2.00E-06
**Transcript_324193**	tubulin alpha chain	-3.36	3.81E-20
**Transcript_332311**	tubulin alpha chain	-2.85	6.61E-12

**Transcript_285898**	tubulin alpha chain	-2.15	3.71E-04
**Transcript_49445**	tubulin alpha-1 chain	-3.02	2.93E-09
**Transcript_327532**	tubulin alpha-1 chain	-2.96	1.46E-12
**Transcript_285897**	tubulin alpha-1 chain	-2.41	1.12E-03
**Transcript_229300**	tubulin alpha-1 chain	-2.04	1.20E-04
**Transcript_207127**	tubulin alpha-1 chain|tubulin alpha-1A chain	-2.23	2.67E-03
**Transcript_306226**	tubulin alpha-1A chain	-5.73	2.00E-03
**Transcript_354200**	tubulin alpha-1A chain	-4.12	8.00E-04
**Transcript_89028**	tubulin alpha-1A chain	-3.17	1.17E-02
**Transcript_367771**	tubulin alpha-1A chain	-3.12	4.06E-05
**Transcript_362085**	tubulin alpha-1A chain	-2.62	1.04E-04
**Transcript_262388**	tubulin alpha-1A chain	-2.56	7.67E-05
**Transcript_377194**	tubulin alpha-1A chain	-2.54	1.27E-06
**Transcript_222321**	tubulin alpha-1A chain	-2.05	5.62E-04
**Transcript_412907**	tubulin alpha-1A chain	-2.04	7.70E-04
**Transcript_323033**	tubulin alpha-2/alpha-4 chain	-2.83	1.99E-02
**Transcript_367619**	tubulin beta chain	-3.62	3.14E-04
**Transcript_277435**	tubulin beta chain	-2.20	2.18E-02
**Transcript_230874**	tyrosine-protein phosphatase non-receptor type 9	-2.10	4.46E-02
**Transcript_196821**	ubiquitin	-2.88	2.92E-02
**Transcript_433726**	UDP-glucuronosyltransferase 2C1|UDP-glucuronosyltransferase 1-2|UDP-glucuronosyltransferase 2A2	-2.73	2.97E-02
**Transcript_402138**	uncharacterized protein LOC100888048	-3.85	4.20E-04
**Transcript_355756**	uncharacterized protein LOC100888517	-2.19	9.35E-05
**Transcript_369230**	uncharacterized protein LOC100891189	-2.61	8.60E-04
**Transcript_40273**	uncharacterized protein LOC105446839	-3.22	6.08E-03
**Transcript_349228**	uncharacterized protein LOC576450	-2.43	1.07E-07
**Transcript_331800**	uncharacterized protein LOC591826	-2.18	1.06E-02
**Transcript_247803**	uncharacterized protein LOC592324	-2.48	6.86E-03
**Transcript_456195**	uncharacterized protein LOC753087|185/333 D1 alpha|185/333 D5 epsilon|185/333 E6 alpha	-2.11	1.73E-04
**Transcript_399035**	uncharacterized protein LOC756005|protein of unknown function DUF2181 containing protein	-3.38	7.19E-08
**Transcript_199904**	uncharacterized protein LOC756131	-2.05	1.38E-04
**Transcript_448557**	vasoactive intestinal polypeptide receptor 1|parathyroid hormone 2 receptor	-2.04	4.20E-03
**Transcript_339985**	von Willebrand factor A domain-containing protein 5B1	-2.40	3.64E-04
**Transcript_223195**	zinc finger SWIM domain-containing protein 8|zinc finger SWIM domain-containing protein KIAA0913-like	-2.01	3.14E-02

*Highlighted transcripts in yellow are nervous system associated genes

Table S9. Differentially expressed transcription factors

ID	Annotated name	Abbreviation	Log2FC	Adjusted p-value
**Transcript_319952**	Transcription factor SOX-4	SOX4	4.50	9.30E-34
**Transcript_263596**	Transcription factor BTF3 homolog 4	BTF3L4	4.16	2.16E-11
**Transcript_175325**	Thyroid transcription factor 1-associated protein 26 homolog	TAP26	3.00	2.98E-11
**Transcript_290528**	Transcription factor VBP	TEF	5.74	1.06E-06
**Transcript_423204**	Forkhead transcription factor A	FOXA	-3.83	3.80E-05
**Transcript_287026**	Transcription factor 25	TCF25	1.77	1.29E-04
**Transcript_443183**	Myc protein	MYC	2.72	2.63E-04
**Transcript_387814**	Transcription factor IIIA	GTF3A	1.86	5.36E-04
**Transcript_239737**	Pre-B-cell leukemia transcription factor 1	PBX3	1.36	1.46E-03
**Transcript_265397**	Transcription factor soxd1	SOXD1	1.43	1.90E-03
**Transcript_373546**	Nuclear transcription factor Y subunit gamma	NFYC	1.35	2.20E-03
**Transcript_244221**	Transcription factor AP-1	AP-1	-1.81	3.44E-03
**Transcript_269278**	Transcription factor HES-4	HES4	-2.55	6.28E-03
**Transcript_286791**	General transcription factor IIH subunit 1	GTF2H1	1.27	1.13E-02
**Transcript_220104**	Homeobox protein Hox-A10	HOXA10	1.55	1.20E-02
**Transcript_321671**	Paired box protein Pax-2a	PAX2A	1.50	1.69E-02
**Transcript_441452**	LIM homeobox transcription factor 1-beta	LMX1B	-1.61	1.83E-02
**Transcript_304792**	ETS-related transcription factor Elf-3	ELF3	1.33	2.47E-02
**Transcript_211042**	Myelin transcription factor 1-like protein	MYT1L1	1.04	2.47E-02
**Transcript_274240**	Beta-catenin	CTNNB	1.03	2.96E-02
**Transcript_224155**	Zinc-finger transcription factor Snail	SNAIL	1.19	9.00E-02
**Transcript_291169**	GATA transcription factor e	GATAE	-1.97	1.20E-01
**Transcript_441904**	Transcription factor E2F5	E2F5	1.01	1.21E-01
**Transcript_39673**	Transcription factor AP-2-alpha	TFAP2A	2.43	1.31E-01
**Transcript_307462**	Homeobox transcription factor Nk1	NK1	-1.93	2.44E-01
**Transcript_361564**	Forkhead transcription factor J1	FOXJ1	-1.08	3.06E-01
**Transcript_107633**	LIM domain transcription factor LMO4-B	LMO4	1.23	3.40E-01
**Transcript_76067**	Winged helix transcription factor Forkhead-1	FKH1	1.05	3.58E-01
**Transcript_168671**	Elongation factor 1 alpha	EEF1A1	7.32	8.38E-05
**Transcript_345735**	Homeobox protein DLX-5|homeobox protein Hox-B4	DLX5	3.07	4.06E-02
**Transcript_429867**	Homeobox protein Hmx	HMX	3.59	8.86E-17
**Transcript_279040**	Krueppel-like factor 13	KLF13	3.21	1.17E-06
**Transcript_433953**	Periodic tryptophan protein 1 homolog	PWP1	3.35	4.80E-12
**Transcript_432532**	Rhombotin-2	LMO2	2.95	1.48E-10
**Transcript_236625**	Homeobox protein CDX-2	CDX1	-8.64	2.33E-03
**Transcript_271521**	Fos-related antigen 1	FOSL2	-4.85	5.48E-03

**Transcript_438891**	Homeobox domain-containing protein	HLX	-4.10	1.38E-17
**Transcript_173387**	Hepatocyte nuclear factor 4-gamma	HNF4G	-3.38	1.70E-03
**Transcript_438353**	Homeobox protein EMX1	EMX1	-4.28	1.09E-03
**Transcript_459460**	THAP domain-containing protein 5	THAP4	2.86	1.08E-02
**Transcript_433507**	Homeobox protein Mohawk-like	MKX	2.61	7.15E-06
**Transcript_284772**	TATA box-binding protein-associated factor RNA polymerase I subunit A	TAF1A	2.54	2.14E-04
**Transcript_189718**	Myb-binding protein 1A-like protein	MYBBP1A	2.38	1.71E-05
**Transcript_459696**	Myoneurin	MYNN	2.11	4.88E-05
**Transcript_268430**	Gastrula zinc finger protein xlcgf57.1 isoform X5	ZNF	-3.06	8.46E-03
**Transcript_247937**	Early growth response protein 1-B	EGR1	-2.75	1.42E-04
**Transcript_330100**	Basic leucine zipper domain, Maf-type	BSL78	-2.45	5.85E-05

Table S10. Gene ontology analysis from day 1 downregulated genes using DAVID

Category	Term	Count	PValue	List.Total	Pop.Hits	Pop.Total	Fold.Enrichment	Bonferroni	Benjamini	FDR	Description
BP	GO:0007155	5	9.67E-04	39	14	1094	10.02	0.31	0.31	1.33	cell adhesion
BP	GO:0022610	5	1.29E-03	39	15	1094	9.35	0.38	0.22	1.76	biological adhesion
BP	GO:0006082	7	1.15E-02	39	57	1094	3.44	0.99	0.58	14.81	organic acid metabolic process
BP	GO:0044699	28	1.97E-02	39	586	1094	1.34	1.00	0.66	24.03	single-organism process
BP	GO:0046394	4	3.34E-02	39	21	1094	5.34	1.00	0.80	37.47	carboxylic acid biosynthetic process
BP	GO:0044763	26	3.49E-02	39	548	1094	1.33	1.00	0.77	38.79	single-organism cellular process
BP	GO:0002376	3	4.44E-02	39	10	1094	8.42	1.00	0.79	46.60	immune system process
BP	GO:0032501	5	4.98E-02	39	41	1094	3.42	1.00	0.80	50.65	multicellular organismal process
BP	GO:0044283	4	6.36E-02	39	27	1094	4.16	1.00	0.85	59.72	small molecule biosynthetic process
CC	GO:0031224	26	6.01E-03	47	445	1253	1.56	0.42	0.42	6.35	intrinsic component of membrane
CC	GO:0016020	29	7.78E-03	47	531	1253	1.46	0.50	0.30	8.15	membrane
CC	GO:0016021	25	1.32E-02	47	444	1253	1.50	0.70	0.33	13.43	integral component of membrane
CC	GO:0044425	26	2.30E-02	47	489	1253	1.42	0.88	0.41	22.35	membrane part
MF	GO:0016798	5	1.02E-03	54	12	1277	9.85	0.16	0.16	1.24	hydrolase activity, acting on glycosyl bonds
MF	GO:0005044	4	3.26E-03	54	8	1277	11.82	0.43	0.24	3.91	scavenger receptor activity
MF	GO:0038024	4	3.26E-03	54	8	1277	11.82	0.43	0.24	3.91	cargo receptor activity
MF	GO:0004872	8	1.19E-02	54	62	1277	3.05	0.87	0.49	13.56	receptor activity
MF	GO:0060089	8	1.19E-02	54	62	1277	3.05	0.87	0.49	13.56	molecular transducer activity
MF	GO:0008237	4	1.78E-02	54	14	1277	6.76	0.95	0.53	19.65	metallopeptidase activity
MF	GO:0003824	34	2.42E-02	54	614	1277	1.31	0.98	0.57	25.83	catalytic activity
MF	GO:0016491	10	2.50E-02	54	105	1277	2.25	0.99	0.51	26.58	oxidoreductase activity
MF	GO:0004553	3	5.05E-02	54	9	1277	7.88	1.00	0.72	46.84	hydrolase activity, hydrolyzing O-glycosyl compounds
MF	GO:0005509	5	8.00E-02	54	40	1277	2.96	1.00	0.83	63.85	calcium ion binding
MF	GO:0016805	2	8.13E-02	54	2	1277	23.65	1.00	0.80	64.47	dipeptidase activity
MF	GO:0003796	2	8.13E-02	54	2	1277	23.65	1.00	0.80	64.47	lysozyme activity
MF	GO:0004497	3	9.84E-02	54	13	1277	5.46	1.00	0.83	71.75	monooxygenase activity

Table S11. Gene ontology analysis from day 1 upregulated genes using DAVID

Category	Term	Count	PValue	List.Total	Pop.Hits	Pop.Total	Fold.Enrichment	Bonferroni	Benjamini	FDR	Description
BP	GO:0022613	23	1.28E-08	99	68	1094	3.74	1.02E-05	1.02E-05	1.97E-05	ribonucleoprotein complex biogenesis
BP	GO:0016072	12	1.52E-05	99	29	1094	4.57	1.21E-02	4.05E-03	2.34E-02	rRNA metabolic process
BP	GO:0034660	15	2.45E-05	99	47	1094	3.53	1.94E-02	4.87E-03	3.76E-02	ncRNA metabolic process
BP	GO:0071840	38	3.45E-04	99	250	1094	1.68	2.41E-01	4.49E-02	5.28E-01	cellular component organization or biogenesis
BP	GO:0010467	41	5.40E-04	99	284	1094	1.60	3.50E-01	5.97E-02	8.25E-01	gene expression
BP	GO:0044085	25	6.48E-04	99	140	1094	1.97	4.04E-01	6.27E-02	9.90E-01	cellular component biogenesis
BP	GO:0006413	6	9.15E-04	99	10	1094	6.63	5.18E-01	7.80E-02	1.40E+00	translational initiation
BP	GO:0034641	53	1.09E-03	99	416	1094	1.41	5.83E-01	8.37E-02	1.67E+00	cellular nitrogen compound metabolic process
BP	GO:0006412	17	1.49E-03	99	82	1094	2.29	6.96E-01	1.02E-01	2.26E+00	translation
BP	GO:0006807	54	2.72E-03	99	441	1094	1.35	8.86E-01	1.44E-01	4.09E+00	nitrogen compound metabolic process
BP	GO:1990542	5	3.20E-03	99	8	1094	6.91	9.23E-01	1.57E-01	4.81E+00	mitochondrial transmembrane transport
BP	GO:0006396	16	3.23E-03	99	80	1094	2.21	9.24E-01	1.49E-01	4.85E+00	RNA processing
BP	GO:1901566	24	4.64E-03	99	151	1094	1.76	9.76E-01	1.86E-01	6.89E+00	organonitrogen compound biosynthetic process
BP	GO:0044260	56	4.67E-03	99	472	1094	1.31	9.76E-01	1.78E-01	6.93E+00	cellular macromolecule metabolic process
BP	GO:1902582	6	5.43E-03	99	14	1094	4.74	9.87E-01	1.95E-01	8.01E+00	single-organism intracellular transport
BP	GO:0043603	18	6.25E-03	99	102	1094	1.95	9.93E-01	2.04E-01	9.18E+00	cellular amide metabolic process
BP	GO:0017038	5	8.34E-03	99	10	1094	5.53	9.99E-01	2.43E-01	1.21E+01	protein import
BP	GO:0006839	5	8.34E-03	99	10	1094	5.53	9.99E-01	2.43E-01	1.21E+01	mitochondrial transport
BP	GO:0043170	56	8.50E-03	99	483	1094	1.28	9.99E-01	2.38E-01	1.23E+01	macromolecule metabolic process
BP	GO:0007005	8	1.12E-02	99	29	1094	3.05	1.00E+00	2.93E-01	1.59E+01	mitochondrion organization
BP	GO:0071826	8	1.12E-02	99	29	1094	3.05	1.00E+00	2.93E-01	1.59E+01	ribonucleoprotein complex subunit organization
BP	GO:0071806	4	1.87E-02	99	7	1094	6.31	1.00E+00	4.16E-01	2.52E+01	protein transmembrane transport
BP	GO:0051246	10	1.96E-02	99	47	1094	2.35	1.00E+00	4.20E-01	2.62E+01	regulation of protein metabolic process
BP	GO:0016070	28	2.24E-02	99	210	1094	1.47	1.00E+00	4.53E-01	2.94E+01	RNA metabolic process
BP	GO:0070585	4	2.80E-02	99	8	1094	5.53	1.00E+00	5.19E-01	3.53E+01	protein localization to mitochondrion
BP	GO:0044267	32	3.19E-02	99	256	1094	1.38	1.00E+00	5.55E-01	3.92E+01	cellular protein metabolic process
BP	GO:0044271	32	3.36E-02	99	257	1094	1.38	1.00E+00	5.63E-01	4.09E+01	cellular nitrogen compound biosynthetic process
BP	GO:0032268	9	3.65E-02	99	44	1094	2.26	1.00E+00	5.82E-01	4.35E+01	regulation of cellular protein metabolic process
BP	GO:0090304	31	4.15E-02	99	251	1094	1.36	1.00E+00	6.20E-01	4.78E+01	nucleic acid metabolic process
BP	GO:0044237	66	4.44E-02	99	632	1094	1.15	1.00E+00	6.25E-01	5.02E+01	cellular metabolic process
BP	GO:0006457	6	4.77E-02	99	23	1094	2.88	1.00E+00	6.41E-01	5.27E+01	protein folding
BP	GO:1901564	25	4.86E-02	99	194	1094	1.42	1.00E+00	6.39E-01	5.34E+01	organonitrogen compound metabolic process
BP	GO:0046483	38	4.97E-02	99	327	1094	1.28	1.00E+00	6.38E-01	5.43E+01	heterocycle metabolic process
BP	GO:0019538	32	5.01E-02	99	265	1094	1.33	1.00E+00	6.32E-01	5.45E+01	protein metabolic process
BP	GO:0006139	37	5.07E-02	99	317	1094	1.29	1.00E+00	6.28E-01	5.50E+01	nucleobase-containing compound metabolic process
BP	GO:0010608	4	5.26E-02	99	10	1094	4.42	1.00E+00	6.33E-01	5.63E+01	posttranscriptional regulation of gene expression
BP	GO:0071704	66	5.62E-02	99	638	1094	1.14	1.00E+00	6.50E-01	5.89E+01	organic substance metabolic process
BP	GO:0006725	38	5.66E-02	99	330	1094	1.27	1.00E+00	6.44E-01	5.91E+01	cellular aromatic compound metabolic process
BP	GO:0044238	63	6.62E-02	99	608	1094	1.15	1.00E+00	6.88E-01	6.51E+01	primary metabolic process
BP	GO:0000469	3	6.64E-02	99	5	1094	6.63	1.00E+00	6.81E-01	6.52E+01	cleavage involved in rRNA processing
BP	GO:0051345	3	6.64E-02	99	5	1094	6.63	1.00E+00	6.81E-01	6.52E+01	positive regulation of hydrolase activity

BP	GO:0009894	4	6.77E-02	99	11	1094	4.02	1.00E+00	6.81E-01	6.59E+01	regulation of catabolic process
BP	GO:0034248	4	6.77E-02	99	11	1094	4.02	1.00E+00	6.81E-01	6.59E+01	regulation of cellular amide metabolic process
BP	GO:1901360	38	6.97E-02	99	335	1094	1.25	1.00E+00	6.77E-01	6.70E+01	organic cyclic compound metabolic process
BP	GO:0090501	3	9.39E-02	99	6	1094	5.53	1.00E+00	7.80E-01	7.80E+01	RNA phosphodiester bond hydrolysis
BP	GO:0000470	3	9.39E-02	99	6	1094	5.53	1.00E+00	7.80E-01	7.80E+01	maturation of LSU-rRNA
BP	GO:0032543	3	9.39E-02	99	6	1094	5.53	1.00E+00	7.80E-01	7.80E+01	mitochondrial translation
CC	GO:0005730	13	1.67E-07	108	25	1253	6.03	3.48E-05	3.48E-05	2.11E-04	nucleolus
CC	GO:0031974	25	8.06E-06	108	113	1253	2.57	1.68E-03	8.38E-04	1.02E-02	membrane-enclosed lumen
CC	GO:0030529	25	1.80E-05	108	118	1253	2.46	3.74E-03	1.25E-03	2.27E-02	intracellular ribonucleoprotein complex
CC	GO:1990904	25	1.80E-05	108	118	1253	2.46	3.74E-03	1.25E-03	2.27E-02	ribonucleoprotein complex
CC	GO:0030684	8	9.92E-05	108	15	1253	6.19	2.04E-02	5.14E-03	1.25E-01	preribosome
CC	GO:0032991	47	8.50E-04	108	363	1253	1.50	1.62E-01	2.50E-02	1.07E+00	macromolecular complex
CC	GO:0044428	22	1.15E-03	108	125	1253	2.04	2.13E-01	2.94E-02	1.44E+00	nuclear part
CC	GO:0043228	28	1.70E-03	108	183	1253	1.78	2.99E-01	3.86E-02	2.13E+00	non-membrane-bounded organelle
CC	GO:0043232	28	1.70E-03	108	183	1253	1.78	2.99E-01	3.86E-02	2.13E+00	intracellular non-membrane-bounded organelle
CC	GO:0044452	5	2.69E-03	108	8	1253	7.25	4.29E-01	5.45E-02	3.34E+00	nucleolar part
CC	GO:0070993	4	5.34E-03	108	5	1253	9.28	6.71E-01	9.62E-02	6.53E+00	translation preinitiation complex
CC	GO:0033290	4	5.34E-03	108	5	1253	9.28	6.71E-01	9.62E-02	6.53E+00	eukaryotic 48S preinitiation complex
CC	GO:0005622	86	1.03E-02	108	869	1253	1.15	8.83E-01	1.64E-01	1.22E+01	intracellular
CC	GO:0005762	4	1.64E-02	108	7	1253	6.63	9.68E-01	2.33E-01	1.89E+01	mitochondrial large ribosomal subunit
CC	GO:0022624	4	1.64E-02	108	7	1253	6.63	9.68E-01	2.33E-01	1.89E+01	proteasome accessory complex
CC	GO:0098798	6	2.27E-02	108	20	1253	3.48	9.92E-01	2.89E-01	2.51E+01	mitochondrial protein complex
CC	GO:0044424	80	2.45E-02	108	812	1253	1.14	9.94E-01	2.91E-01	2.69E+01	intracellular part
CC	GO:0043234	32	2.72E-02	108	264	1253	1.41	9.97E-01	3.01E-01	2.93E+01	protein complex
CC	GO:0005634	36	2.96E-02	108	308	1253	1.36	9.98E-01	3.07E-01	3.15E+01	nucleus
CC	GO:0005623	91	3.12E-02	108	959	1253	1.10	9.99E-01	3.07E-01	3.30E+01	cell
CC	GO:0005852	3	3.87E-02	108	4	1253	8.70	1.00E+00	3.36E-01	3.92E+01	eukaryotic translation initiation factor 3 complex
CC	GO:0005615	6	5.52E-02	108	25	1253	2.78	1.00E+00	4.15E-01	5.12E+01	extracellular space
CC	GO:0005732	3	6.09E-02	108	5	1253	6.96	1.00E+00	4.33E-01	5.47E+01	small nucleolar ribonucleoprotein complex
CC	GO:0005739	15	6.72E-02	108	108	1253	1.61	1.00E+00	4.53E-01	5.84E+01	mitochondrion
CC	GO:0044422	40	8.38E-02	108	377	1253	1.23	1.00E+00	5.03E-01	6.68E+01	organelle part
CC	GO:0032040	3	8.63E-02	108	6	1253	5.80	1.00E+00	5.01E-01	6.80E+01	small-subunit processome
CC	GO:0005758	3	8.63E-02	108	6	1253	5.80	1.00E+00	5.01E-01	6.80E+01	mitochondrial intermembrane space
CC	GO:0031970	3	8.63E-02	108	6	1253	5.80	1.00E+00	5.01E-01	6.80E+01	organelle envelope lumen
MF	GO:0003723	21	1.85E-06	98	86	1277	3.18	4.64E-04	4.64E-04	2.40E-03	RNA binding
MF	GO:0008135	10	8.01E-06	98	21	1277	6.21	2.01E-03	1.00E-03	1.04E-02	translation factor activity, RNA binding
MF	GO:0003743	7	4.71E-04	98	15	1277	6.08	1.11E-01	3.86E-02	6.10E-01	translation initiation factor activity
MF	GO:0008565	4	1.80E-02	98	8	1277	6.52	9.89E-01	6.80E-01	2.10E+01	protein transporter activity
MF	GO:0003676	29	4.59E-02	98	274	1277	1.38	1.00E+00	9.05E-01	4.57E+01	nucleic acid binding
MF	GO:1901363	48	5.68E-02	98	515	1277	1.21	1.00E+00	9.13E-01	5.32E+01	heterocyclic compound binding
MF	GO:0097159	48	6.47E-02	98	519	1277	1.21	1.00E+00	9.09E-01	5.81E+01	organic cyclic compound binding
MF	GO:0034062	3	7.00E-02	98	6	1277	6.52	1.00E+00	8.98E-01	6.11E+01	RNA polymerase activity
MF	GO:0003899	3	7.00E-02	98	6	1277	6.52	1.00E+00	8.98E-01	6.11E+01	DNA-directed RNA polymerase activity
MF	GO:0016779	4	8.38E-02	98	14	1277	3.72	1.00E+00	9.13E-01	6.79E+01	nucleotidyltransferase activity

Table S12. Gene ontology analysis from day 3 downregulated genes using DAVID

Category	Term	Count	PValue	List.Total	Pop.Hits	Pop.Total	Fold.Enrichment	Bonferroni	Benjamini	FDR	Description
BP	GO:0099537	3	3.22E-02	33	10	1094	9.95	1.00	1.00	35.00	trans-synaptic signaling
BP	GO:0098916	3	3.22E-02	33	10	1094	9.95	1.00	1.00	35.00	anterograde trans-synaptic signaling
BP	GO:0099536	3	3.22E-02	33	10	1094	9.95	1.00	1.00	35.00	synaptic signaling
BP	GO:0007268	3	3.22E-02	33	10	1094	9.95	1.00	1.00	35.00	chemical synaptic transmission
BP	GO:0007186	4	4.13E-02	33	27	1094	4.91	1.00	1.00	42.55	G-protein coupled receptor signaling pathway
BP	GO:0007154	10	5.60E-02	33	175	1094	1.89	1.00	0.99	53.11	cell communication
BP	GO:0044699	23	5.70E-02	33	586	1094	1.30	1.00	0.98	53.80	single-organism process
BP	GO:0007155	3	6.06E-02	33	14	1094	7.10	1.00	0.97	56.04	cell adhesion
BP	GO:0022610	3	6.87E-02	33	15	1094	6.63	1.00	0.96	60.76	biological adhesion
CC	GO:0031224	33	1.77E-06	47	445	1253	1.98	0.00	0.00	0.00	intrinsic component of membrane
CC	GO:0016021	32	7.07E-06	47	444	1253	1.92	0.00	0.00	0.01	integral component of membrane
CC	GO:0016020	35	1.02E-05	47	531	1253	1.76	0.00	0.00	0.01	membrane
CC	GO:0031226	12	1.35E-05	47	67	1253	4.77	0.00	0.00	0.02	intrinsic component of plasma membrane
CC	GO:0005887	12	1.35E-05	47	67	1253	4.77	0.00	0.00	0.02	integral component of plasma membrane
CC	GO:0044425	33	1.91E-05	47	489	1253	1.80	0.00	0.00	0.02	membrane part
CC	GO:0071944	14	3.05E-05	47	101	1253	3.70	0.00	0.00	0.04	cell periphery
CC	GO:0044459	12	5.36E-05	47	77	1253	4.15	0.01	0.00	0.06	plasma membrane part
CC	GO:0005886	13	1.11E-04	47	98	1253	3.54	0.02	0.00	0.13	plasma membrane
CC	GO:0005576	5	4.71E-02	47	38	1253	3.51	1.00	0.58	44.16	extracellular region
MF	GO:0060089	12	2.23E-05	54	62	1277	4.58	0.00	0.00	0.03	molecular transducer activity
MF	GO:0004872	12	2.23E-05	54	62	1277	4.58	0.00	0.00	0.03	receptor activity
MF	GO:0004871	8	1.19E-02	54	62	1277	3.05	0.89	0.31	13.74	signal transducer activity
MF	GO:0016491	10	2.50E-02	54	105	1277	2.25	0.99	0.49	26.90	oxidoreductase activity
MF	GO:0005044	3	4.03E-02	54	8	1277	8.87	1.00	0.61	39.90	scavenger receptor activity
MF	GO:0038024	3	4.03E-02	54	8	1277	8.87	1.00	0.61	39.90	cargo receptor activity
MF	GO:1901618	2	8.13E-02	54	2	1277	23.65	1.00	0.83	64.99	organic hydroxy compound transmembrane transporter activity
MF	GO:0052689	3	8.55E-02	54	12	1277	5.91	1.00	0.81	66.91	carboxylic ester hydrolase activity
MF	GO:0004497	3	9.84E-02	54	13	1277	5.46	1.00	0.82	72.26	monooxygenase activity

Table S13. Gene ontology analysis from day 3 Upregulated genes using DAVID

Category	Term	Count	PValue	List.Total	Pop.Hits	Pop.Total	Fold.Enrichment	Bonferroni	Benjamini	FDR	Description
BP	GO:0051246	9	7.48E-04	50	47	1094	4.19	0.35	0.35	1.10	regulation of protein metabolic process
BP	GO:0032268	8	2.51E-03	50	44	1094	3.98	0.77	0.52	3.63	regulation of cellular protein metabolic process
BP	GO:0006396	10	6.99E-03	50	80	1094	2.74	0.98	0.64	9.80	RNA processing
BP	GO:0034470	7	9.04E-03	50	42	1094	3.65	0.99	0.65	12.50	ncRNA processing
BP	GO:0009894	4	1.08E-02	50	11	1094	7.96	1.00	0.65	14.78	regulation of catabolic process
BP	GO:0034660	7	1.56E-02	50	47	1094	3.26	1.00	0.68	20.61	ncRNA metabolic process
BP	GO:0006457	5	1.68E-02	50	23	1094	4.76	1.00	0.67	22.02	protein folding
BP	GO:0000470	3	2.63E-02	50	6	1094	10.94	1.00	0.79	32.40	maturation of LSU-rRNA
BP	GO:0022613	8	2.77E-02	50	68	1094	2.57	1.00	0.77	33.86	ribonucleoprotein complex biogenesis
BP	GO:0044260	29	3.08E-02	50	472	1094	1.34	1.00	0.78	36.91	cellular macromolecule metabolic process
BP	GO:0071806	3	3.57E-02	50	7	1094	9.38	1.00	0.80	41.45	protein transmembrane transport
BP	GO:0065002	3	3.57E-02	50	7	1094	9.38	1.00	0.80	41.45	intracellular protein transmembrane transport
BP	GO:0042273	4	3.69E-02	50	17	1094	5.15	1.00	0.79	42.46	ribosomal large subunit biogenesis
BP	GO:0043170	29	4.25E-02	50	483	1094	1.31	1.00	0.79	47.24	macromolecule metabolic process
BP	GO:0044267	18	4.52E-02	50	256	1094	1.54	1.00	0.79	49.33	cellular protein metabolic process
BP	GO:0006626	3	4.63E-02	50	8	1094	8.21	1.00	0.78	50.21	protein targeting to mitochondrion
BP	GO:0009451	4	4.94E-02	50	19	1094	4.61	1.00	0.79	52.50	RNA modification
BP	GO:0010467	19	5.89E-02	50	284	1094	1.46	1.00	0.81	59.08	gene expression
BP	GO:0019538	18	6.08E-02	50	265	1094	1.49	1.00	0.81	60.25	protein metabolic process
BP	GO:0048522	6	6.30E-02	50	49	1094	2.68	1.00	0.81	61.61	positive regulation of cellular process
BP	GO:0048518	6	6.30E-02	50	49	1094	2.68	1.00	0.81	61.61	positive regulation of biological process
BP	GO:0006412	8	6.70E-02	50	82	1094	2.13	1.00	0.80	63.95	translation
BP	GO:0051336	3	7.03E-02	50	10	1094	6.56	1.00	0.80	65.77	regulation of hydrolase activity
BP	GO:0042981	3	7.03E-02	50	10	1094	6.56	1.00	0.80	65.77	regulation of apoptotic process
BP	GO:0006839	3	7.03E-02	50	10	1094	6.56	1.00	0.80	65.77	mitochondrial transport
BP	GO:0010608	3	7.03E-02	50	10	1094	6.56	1.00	0.80	65.77	posttranscriptional regulation of gene expression
BP	GO:0017038	3	7.03E-02	50	10	1094	6.56	1.00	0.80	65.77	protein import
BP	GO:0006413	3	7.03E-02	50	10	1094	6.56	1.00	0.80	65.77	translational initiation
BP	GO:0034641	25	7.27E-02	50	416	1094	1.31	1.00	0.79	67.04	cellular nitrogen compound metabolic process
BP	GO:0043603	9	7.74E-02	50	102	1094	1.93	1.00	0.80	69.42	cellular amide metabolic process
BP	GO:0006807	26	7.95E-02	50	441	1094	1.29	1.00	0.80	70.43	nitrogen compound metabolic process
BP	GO:0034248	3	8.35E-02	50	11	1094	5.97	1.00	0.81	72.27	regulation of cellular amide metabolic process
BP	GO:0044085	11	8.43E-02	50	140	1094	1.72	1.00	0.80	72.60	cellular component biogenesis
BP	GO:0030155	2	8.76E-02	50	2	1094	21.88	1.00	0.80	74.04	regulation of cell adhesion
BP	GO:0030334	2	8.76E-02	50	2	1094	21.88	1.00	0.80	74.04	regulation of cell migration
BP	GO:0032231	2	8.76E-02	50	2	1094	21.88	1.00	0.80	74.04	regulation of actin filament bundle assembly
CC	GO:0030529	16	1.24E-04	58	118	1253	2.93	0.02	0.02	0.15	intracellular ribonucleoprotein complex
CC	GO:1990904	16	1.24E-04	58	118	1253	2.93	0.02	0.02	0.15	ribonucleoprotein complex
CC	GO:0005615	7	6.15E-04	58	25	1253	6.05	0.09	0.05	0.74	extracellular space

CC	GO:0032991	29	8.02E-04	58	363	1253	1.73	0.12	0.04	0.97	macromolecular complex
CC	GO:0022624	4	2.75E-03	58	7	1253	12.34	0.36	0.09	3.27	proteasome accessory complex
CC	GO:0005730	6	4.31E-03	58	25	1253	5.18	0.50	0.11	5.09	nucleolus
CC	GO:0005576	7	6.04E-03	58	38	1253	3.98	0.62	0.13	7.06	extracellular region
CC	GO:0005732	3	1.86E-02	58	5	1253	12.96	0.95	0.29	20.34	small nucleolar ribonucleoprotein complex
CC	GO:0005758	3	2.71E-02	58	6	1253	10.80	0.99	0.36	28.29	mitochondrial intermembrane space
CC	GO:0031970	3	2.71E-02	58	6	1253	10.80	0.99	0.36	28.29	organelle envelope lumen
CC	GO:0030684	4	2.75E-02	58	15	1253	5.76	0.99	0.34	28.65	preribosome
CC	GO:0043228	15	2.91E-02	58	183	1253	1.77	0.99	0.33	30.01	non-membrane-bounded organelle
CC	GO:0043232	15	2.91E-02	58	183	1253	1.77	0.99	0.33	30.01	intracellular non-membrane-bounded organelle
CC	GO:0044424	44	5.45E-02	58	812	1253	1.17	1.00	0.50	49.23	intracellular part
CC	GO:0031974	10	6.36E-02	58	113	1253	1.91	1.00	0.53	54.83	membrane-enclosed lumen
CC	GO:0043234	18	7.16E-02	58	264	1253	1.47	1.00	0.55	59.29	protein complex
CC	GO:0031429	2	8.89E-02	58	2	1253	21.60	1.00	0.61	67.61	box H/ACA snoRNP complex
CC	GO:0042555	2	8.89E-02	58	2	1253	21.60	1.00	0.61	67.61	MCM complex
CC	GO:0005697	2	8.89E-02	58	2	1253	21.60	1.00	0.61	67.61	telomerase holoenzyme complex
MF	GO:0003723	9	7.28E-03	45	86	1277	2.97	0.65	0.65	8.30	RNA binding
MF	GO:0003676	15	6.90E-02	45	274	1277	1.55	1.00	0.99	57.15	nucleic acid binding
MF	GO:0097159	24	7.56E-02	45	519	1277	1.31	1.00	0.98	60.61	organic cyclic compound binding
MF	GO:0061135	2	9.99E-02	45	3	1277	18.92	1.00	0.98	71.30	endopeptidase regulator activity
MF	GO:0061134	2	9.99E-02	45	3	1277	18.92	1.00	0.98	71.30	peptidase regulator activity

Table S14. Pathway enrichment analysis from day 1 downregulated genes using KEGG database

KEGG ID	Pathway	N	Count	Pvalue	GeneRatio
path:spu00010	Glycolysis / Gluconeogenesis	53	5	4.61E-03	9.43E-02
path:spu00040	Pentose and glucuronate interconversions	85	5	3.13E-02	5.88E-02
path:spu00053	Ascorbate and aldarate metabolism	85	5	3.13E-02	5.88E-02
path:spu00071	Fatty acid degradation	57	4	3.01E-02	7.02E-02
path:spu00250	Alanine, aspartate and glutamate metabolism	36	3	3.81E-02	8.33E-02
path:spu00270	Cysteine and methionine metabolism	70	5	1.47E-02	7.14E-02
path:spu00280	Valine, leucine and isoleucine degradation	61	6	1.55E-03	9.84E-02
path:spu00340	Histidine metabolism	37	4	6.91E-03	1.08E-01
path:spu00350	Tyrosine metabolism	28	3	1.97E-02	1.07E-01
path:spu00380	Tryptophan metabolism	74	6	4.15E-03	8.11E-02
path:spu00410	beta-Alanine metabolism	30	3	2.37E-02	1.00E-01
path:spu00620	Pyruvate metabolism	43	5	1.82E-03	1.16E-01
path:spu00730	Thiamine metabolism	17	2	4.77E-02	1.18E-01
path:spu00760	Nicotinate and nicotinamide metabolism	40	3	4.97E-02	7.50E-02
path:spu00790	Folate biosynthesis	48	6	4.25E-04	1.25E-01
path:spu00920	Sulfur metabolism	20	4	6.64E-04	2.00E-01
path:spu01100	Metabolic pathways	1996	66	7.75E-07	3.31E-02
path:spu01200	Carbon metabolism	138	7	2.42E-02	5.07E-02
path:spu04146	Peroxisome	146	9	3.14E-03	6.16E-02
path:spu04512	ECM-receptor interaction	50	4	1.96E-02	8.00E-02

Table S15. Pathway enrichment analysis from day 1 Upregulated genes using KEGG database

KEGG ID	Pathway	N	Count	Pvalue	GeneRatio
path:spu00970	Aminoacyl-tRNA biosynthesis	74	10	1.96E-03	1.35E-01
path:spu03008	Ribosome biogenesis in eukaryotes	91	26	1.41E-14	2.86E-01
path:spu03010	Ribosome	140	30	5.25E-13	2.14E-01
path:spu03013	RNA transport	170	16	5.02E-03	9.41E-02
path:spu03015	mRNA surveillance pathway	72	7	4.76E-02	9.72E-02
path:spu03020	RNA polymerase	31	7	4.21E-04	2.26E-01
path:spu03040	Spliceosome	177	14	3.38E-02	7.91E-02
path:spu03050	Proteasome	48	15	1.70E-09	3.13E-01
path:spu04141	Protein processing in endoplasmic reticulum	166	13	4.28E-02	7.83E-02

Table S16. Pathway enrichment analysis from day 3 downgulated genes using KEGG database

KEGG ID	Pathway	N	Count	Pvalue	GeneRatio
path:spu00340	Histidine metabolism	37	3	2.58E-02	8.11E-02
path:spu00380	Tryptophan metabolism	74	4	3.90E-02	5.41E-02
path:spu00500	Starch and sucrose metabolism	39	3	2.96E-02	7.69E-02
path:spu00620	Pyruvate metabolism	43	3	3.80E-02	6.98E-02
path:spu00670	One carbon pool by folate	17	2	3.43E-02	1.18E-01
path:spu00730	Thiamine metabolism	17	2	3.43E-02	1.18E-01
path:spu01100	Metabolic pathways	1996	43	4.28E-02	2.15E-02
path:spu04145	Phagosome	165	11	1.14E-04	6.67E-02
path:spu04146	Peroxisome	146	7	1.30E-02	4.79E-02

Table S17. Pathway enrichment analysis from day 3 upgulated genes using KEGG database

KEGG ID	Pathway	N	Count	Pvalue	GeneRatio
path:spu03008	Ribosome biogenesis in eukaryotes	91	7	4.94E-03	7.69E-02
path:spu03010	Ribosome	140	24	3.89E-15	1.71E-01
path:spu03050	Proteasome	48	9	1.14E-06	1.88E-01
path:spu04141	Protein processing in endoplasmic reticulum	166	13	1.06E-04	7.83E-02
path:spu04145	Phagosome	165	8	3.73E-02	4.85E-02

Table S18. Clusters of coexpressed transcripts

ID	Description	Cluster	Adjusted P-value	GeneID
Transcript_442613	catalase	1	5.41E-42	548621
Transcript_268943	cytochrome P450 2U1	2	4.53E-38	581885
Transcript_319952	SOX4	2	9.30E-34	593520
Transcript_285077	zinc transporter ZIP14	2	5.19E-31	763759
Transcript_340144	uncharacterized protein K02A2.6-like|Reverse transcriptase domain-containing protein	2	9.49E-30	105446129
Transcript_328691	MAM and LDL-receptor class A domain-containing protein 1	1	7.43E-27	574589
Transcript_298293	collagen alpha-1(XII) chain|tenascin-X	1	2.46E-26	763365
Transcript_197637	retinol dehydrogenase 12|dehydrogenase/reductase SDR family member 13-like	3	7.29E-26	589120
Transcript_204404	laminin subunit alpha|laminin alpha chain	1	2.41E-25	578626
Transcript_298730	laminin subunit beta-1|laminin, beta 1	4	2.59E-24	582206
Transcript_202375	fibrillin-2|uncharacterized protein LOC105436405	3	4.08E-24	105436405
Transcript_287164	nucleolar complex protein 2 homolog	3	8.11E-24	585237
Transcript_243955	glycerol kinase	3	2.87E-23	580504
Transcript_445893	ATPase family AAA domain-containing protein 3-B	3	5.85E-23	588531
Transcript_298217	4-aminobutyrate aminotransferase, mitochondrial	4	4.79E-22	577656
Transcript_264429	sushi domain-containing protein 2|uncharacterized protein K03H1.5	1	4.74E-21	583122
Transcript_172789	neuroblast differentiation-associated protein AHNAK-like	1	1.09E-20	105441784
Transcript_442463	probable dimethyladenosine transferase	2	1.72E-20	574553
Transcript_280127	RNA granule protein invertebrate|TPA: RNA granule protein invertebrate	2	3.94E-20	580088
Transcript_406209	sulfite oxidase	4	7.13E-20	586173
Transcript_238520	lysyl oxidase-like 2	1	5.75E-19	585092
Transcript_267297	pumilio homolog 3	3	7.79E-19	583455
Transcript_443315	prestalk protein	1	1.77E-18	590178
Transcript_461742	stromelysin-3-like|matrix metalloproteinase-24-like	1	2.08E-18	105436437
Transcript_385039	aminopeptidase N	1	2.21E-18	578352
Transcript_438891	HLX	5	1.38E-17	100892685
Transcript_350383	telomerase reverse transcriptase-long|telomerase reverse transcriptase-	3	2.59E-17	105447117
Transcript_344692	26S proteasome non-ATPase regulatory subunit 12	3	2.62E-17	593806
Transcript_429867	HMX	3	8.86E-17	579532
Transcript_395263	26S proteasome non-ATPase regulatory subunit 5	2	1.55E-16	588345
Transcript_198009	uncharacterized protein LOC582064|inter-alpha-trypsin inhibitor heavy chain H2	4	2.22E-16	582064
Transcript_246166	diphthine synthase	3	2.57E-16	577892

Transcript_430669	LOW QUALITY PROTEIN: uncharacterized protein LOC580672|apolipoprotein B-100	4	2.78E-16	580672
Transcript_328858	uncharacterized protein LOC100893633|Arylamine N-acetyltransferase	4	2.78E-16	100893633
Transcript_414587	solute carrier family 35 member F2	2	6.89E-16	574685
Transcript_364016	prohibitin	2	7.21E-16	584483
Transcript_431156	GTP-binding protein 8	1	8.05E-16	590744
Transcript_366520	GDP-mannose 4,6 dehydratase	2	1.21E-15	588885
Transcript_288662	microfibril-associated glycoprotein 4|tenascin-N	1	1.84E-15	753235
Transcript_351600	peroxisomal multifunctional enzyme type 2|hydroxysteroid (17-beta) dehydrogenase 4	1	2.44E-15	581580
Transcript_267740	ribosome biogenesis protein WDR12	2	3.19E-15	591474
Transcript_404053	mRNA turnover protein 4 homolog	3	3.39E-15	578804
Transcript_364345	proteasome activator complex subunit 3	3	4.11E-15	585921
Transcript_461114	yrdC domain-containing protein, mitochondrial	2	4.36E-15	588740
Transcript_204651	OTU domain-containing protein 4|putative bifunctional UDP-N-acetylglucosamine transferase a	3	6.67E-15	593098
Transcript_425984	translocator protein	1	7.60E-15	583617
Transcript_179579	methenyltetrahydrofolate synthase domain-containing protein	3	8.49E-15	588795
Transcript_183015	LOW QUALITY PROTEIN: histidine--tRNA ligase, cytoplasmic	3	1.13E-14	105444109
Transcript_206897	actin, muscle	1	1.22E-14	581500
Transcript_421726	ribosome production factor 2 homolog	2	1.23E-14	755131
Transcript_365697	uncharacterized protein LOC589195	3	1.28E-14	589195
Transcript_428650	ammonium transporter Rh type B|ammonium transporter Rh type B-A|ammonium transporter	1	1.34E-14	584796
Transcript_431326	26S proteasome non-ATPase regulatory subunit 3	3	1.41E-14	578762
Transcript_384474	transmembrane protein KIAA1109	4	1.46E-14	586038
Transcript_365782	LOW QUALITY PROTEIN: eukaryotic translation initiation factor 3 subunit I	2	2.21E-14	589571
Transcript_279405	ATP-dependent RNA helicase HAS1|ATP-dependent RNA helicase DDX18|DEAD (Asp-Glu-Ala-As	3	2.62E-14	577986
Transcript_197448	negative elongation factor C/D|negative elongation factor D	2	3.61E-14	581078
Transcript_375637	protein Wnt-6	3	6.03E-14	585147
Transcript_344765	guanine nucleotide-binding protein-like NSN1|guanine nucleotide-binding protein-like 3 homol	2	7.57E-14	577855
Transcript_354584	enolase|alpha-enolase-like	1	7.95E-14	579256
Transcript_423794	activator of 90 kDa heat shock protein ATPase homolog 1	2	1.22E-13	575643
Transcript_359489	eukaryotic translation initiation factor 3 subunit K	3	1.62E-13	587575
Transcript_397594	LOW QUALITY PROTEIN: RNA cytidine acetyltransferase|N-acetyltransferase 10 (GCN5-related)	2	1.63E-13	576671
Transcript_373223	RNA polymerase II subunit A C-terminal domain phosphatase SSU72	2	1.68E-13	574857
Transcript_460570	alpha-catulin	1	1.81E-13	576107
Transcript_417350	ribosome biogenesis protein NOP53|glioma tumor suppressor candidate region gene 2 protein	2	1.98E-13	593605
Transcript_333973	tubulin polymerization-promoting protein family member 2	1	2.23E-13	577151
Transcript_183358	alpha-actinin|alpha-actinin, sarcomeric	1	2.26E-13	592971

Transcript_270919	heterogeneous nuclear ribonucleoprotein C	3	2.92E-13	100893477
Transcript_263624	protein Wnt-9a	3	3.13E-13	575320
Transcript_298954	uncharacterized protein LOC577534	2	3.22E-13	577534
Transcript_202461	opsin Rh5	2	4.05E-13	579899
Transcript_427877	uncharacterized protein LOC593232|Ankyrin repeat-containing domain-containing protein	3	4.30E-13	593232
Transcript_375646	hydroxyacylglutathione hydrolase, mitochondrial	1	5.05E-13	578596
Transcript_373821	mitochondrial intermembrane space import and assembly protein 40	2	5.98E-13	582540
Transcript_256657	carbohydrate sulfotransferase 1	2	7.10E-13	579726
Transcript_412104	probable cation-transporting ATPase 13A3|ATPase type 13A3|putative cation-transporting ATP	1	7.52E-13	577790
Transcript_314464	CMP-N-acetylneuraminate-beta-1,4-galactoside alpha-2,3-sialyltransferase	1	8.96E-13	754196
Transcript_359719	heat shock cognate 71 kDa protein|heat shock 70kDa protein 8	2	9.55E-13	576276
Transcript_395874	U3 small nucleolar RNA-associated protein 18 homolog	3	9.76E-13	579700
Transcript_405927	NADPH oxidase 4	2	1.11E-12	100889322
Transcript_448868	IgGFc-binding protein|Fc fragment of IgG binding protein|zonadhesin	1	1.14E-12	762415
Transcript_271439	T-complex protein 1 subunit zeta	2	1.41E-12	577393
Transcript_330216	aspartyl/asparaginyl beta-hydroxylase|bromodomain-containing protein 4	1	1.92E-12	593797
Transcript_200469	aquaporin-8	1	2.12E-12	589852
Transcript_197881	eukaryotic translation initiation factor 3 subunit E	2	2.28E-12	754815
Transcript_182900	sodium-coupled monocarboxylate transporter 1	1	2.33E-12	580845
Transcript_396851	soluble adenylyl cyclase	3	2.73E-12	574069
Transcript_383783	bone morphogenetic protein 1 homolog	1	2.81E-12	373360
Transcript_431169	T-complex protein 1 subunit theta	3	3.11E-12	583086
Transcript_443125	nucleolar RNA helicase 2	2	3.20E-12	581409
Transcript_277158	ATP-binding cassette sub-family F member 1	2	3.40E-12	576400
Transcript_249697	lupus La protein homolog	2	3.41E-12	105443896
Transcript_442985	asparagine synthetase [glutamine-hydrolyzing]	2	3.42E-12	575946
Transcript_262723	probable serine/threonine-protein kinase DDB_G0278665	1	4.05E-12	577336
Transcript_225361	contactin-associated protein-like 2|contactin-associated protein-like 5	1	4.57E-12	575215
Transcript_433953	PWP1	2	4.80E-12	583947
Transcript_353989	nidogen-1	1	5.18E-12	586685
Transcript_350978	ribosome biogenesis regulatory protein homolog	3	5.94E-12	575632
Transcript_294196	low-density lipoprotein receptor-related protein 1	1	8.65E-12	582052
Transcript_278301	tubulin alpha-1A chain	2	9.88E-12	754102
Transcript_258721	glutamyl aminopeptidase	1	1.03E-11	587683
Transcript_440792	medium-chain acyl-CoA ligase ACSF2, mitochondrial|acyl-CoA synthetase family member 2, mit	1	1.35E-11	592091
Transcript_394161	pyrroline-5-carboxylate reductase 1, mitochondrial	2	1.39E-11	583853

Transcript_267275	prohibitin-2	3	1.70E-11	575755
Transcript_263596	BTF3L4	2	2.16E-11	586588
Transcript_267873	H/ACA ribonucleoprotein complex subunit 1	3	2.51E-11	592267
Transcript_385986	midasin	3	2.92E-11	575173
Transcript_336637	eukaryotic peptide chain release factor GTP-binding subunit ERF3A|G1 to S phase transition 2	3	2.92E-11	580307
Transcript_175325	TAP26	2	2.98E-11	579591
Transcript_310211	protein arginine N-methyltransferase 1	3	3.24E-11	590987
Transcript_230511	ribosomal RNA processing protein 1 homolog A|ribosomal RNA processing protein 1 homolog B	2	3.35E-11	594061
Transcript_68378	uncharacterized protein LOC584236	2	3.50E-11	584236
Transcript_452859	25S rRNA (cytosine-C(5))-methyltransferase nop2	3	3.62E-11	582075
Transcript_352347	RNA-binding protein with serine-rich domain 1	3	3.88E-11	585553
Transcript_354900	mitochondrial import receptor subunit TOM40 homolog	3	3.95E-11	585934
Transcript_365226	protein phosphatase 1G	2	4.53E-11	594091
Transcript_296057	carbohydrate sulfotransferase 15-like	1	4.53E-11	100892795
Transcript_222572	bone morphogenetic protein BMP2/4	3	4.69E-11	373196
Transcript_216724	uncharacterized protein LOC587705|putative sterigmatocystin biosynthesis dehydrogenase stc	1	4.87E-11	587705
Transcript_311413	amassin|coelomocyte amassing protein	3	4.95E-11	373503
Transcript_263997	mitochondrial import inner membrane translocase subunit Tim17-B	3	5.59E-11	577928
Transcript_242486	polycomb protein eed-A	3	5.77E-11	581239
Transcript_454730	LOW QUALITY PROTEIN: allene oxide synthase-lipoxygenase protein	1	6.46E-11	584481
Transcript_279511	mucosa-associated lymphoid tissue lymphoma translocation protein 1|mucosa-associated lymp	2	6.81E-11	590194
Transcript_322705	THO complex subunit 6 homolog	3	6.98E-11	580431
Transcript_421292	sphingosine-1-phosphate lyase 1	3	8.18E-11	585643
Transcript_226274	uncharacterized protein LOC583933	1	8.46E-11	583933
Transcript_435175	sodium- and chloride-dependent neutral and basic amino acid transporter B(0+)	1	9.08E-11	594544
Transcript_224605	ribosomal RNA small subunit methyltransferase NEP1	2	9.38E-11	589275
Transcript_342811	epoxide hydrolase 1	1	9.89E-11	588166
Transcript_328958	H/ACA ribonucleoprotein complex subunit DKC1|dyskeratosis congenita 1, dyskerin	2	1.10E-10	584530
Transcript_421302	equilibrative nucleoside transporter 1|microtubule-associated protein 10	4	1.13E-10	592207
Transcript_339471	fibrillin-1	1	1.23E-10	575027
Transcript_431258	methanethiol oxidase|selenium-binding protein 1	1	1.23E-10	582523
Transcript_7357	cathepsin Z	2	1.37E-10	580617
Transcript_432532	LMO2	2	1.48E-10	100892046
Transcript_310947	rRNA-processing protein FCF1 homolog	3	1.49E-10	586103
Transcript_407052	C3 and PZP-like alpha-2-macroglobulin domain-containing protein 8|C3 and PZP-like, alpha-2-m	1	1.50E-10	579831
Transcript_461041	eEF1A lysine and N-terminal methyltransferase|methyltransferase-like protein 13	2	1.67E-10	754600

Transcript_205415	H/ACA ribonucleoprotein complex subunit 2-like protein	3	1.73E-10	577044
Transcript_254399	vacuolar protein sorting-associated protein 13A|vacuolar protein sorting-associated protein 13[illegible]	1	1.75E-10	575234
Transcript_439620	uridine-cytidine kinase 2|uridine-cytidine kinase 2-B	2	1.86E-10	588945
Transcript_208693	neutral and basic amino acid transport protein rBAT	1	1.88E-10	586832
Transcript_395337	formin-J	3	2.03E-10	588660
Transcript_184579	RWD domain-containing protein 1	2	2.11E-10	590636
Transcript_282088	39S ribosomal protein L17, mitochondrial	2	2.40E-10	591498
Transcript_271370	rRNA 2'-O-methyltransferase fibrillarin	3	2.46E-10	752335
Transcript_417755	transmembrane protein 69	2	2.59E-10	586174
Transcript_409638	heat shock 70 kDa protein 14	3	2.73E-10	588149
Transcript_259246	pre-mRNA-splicing factor 38B	2	3.02E-10	100889783
Transcript_308724	uncharacterized protein LOC577145	1	3.19E-10	577145
Transcript_330347	stress-70 protein, mitochondrial|heat shock 70kDa protein 9 (mortalin)	3	3.20E-10	577721
Transcript_215804	leucine-rich repeat-containing protein 74B	1	3.42E-10	584206
Transcript_204158	cystine/glutamate transporter	4	3.52E-10	579243
Transcript_243187	DNA-directed RNA polymerase I subunit RPA49	2	3.56E-10	588897
Transcript_290985	glycine--tRNA ligase	2	4.15E-10	578936
Transcript_403536	ribosome production factor 1	2	4.37E-10	592162
Transcript_295742	10 kDa heat shock protein, mitochondrial	3	4.72E-10	762428
Transcript_197679	uncharacterized protein LOC105446114	4	4.73E-10	105446114
Transcript_456681	CD82 antigen|carbohydrate sulfotransferase 1-like	1	4.92E-10	100889350
Transcript_263817	eukaryotic translation initiation factor 2 subunit 1	3	4.98E-10	586283
Transcript_357723	translation elongation factor 1B beta subunit	3	6.19E-10	574071
Transcript_234345	bromodomain-containing protein 4-like|dentin sialophosphoprotein	3	6.36E-10	105441791
Transcript_270485	U3 small nucleolar RNA-interacting protein 2	2	6.67E-10	582919
Transcript_202897	dimethyladenosine transferase 2, mitochondrial	2	6.97E-10	589505
Transcript_427783	uncharacterized protein LOC575162	1	7.09E-10	575162
Transcript_319739	carboxypeptidase B	1	7.54E-10	764971
Transcript_183115	nucleolar GTP-binding protein 2	2	8.38E-10	585370
Transcript_367031	ribosome biogenesis protein bop1-B	2	8.81E-10	575063
Transcript_272225	protein ABHD11|alpha/beta hydrolase domain-containing protein 11	3	8.95E-10	575326
Transcript_288791	ATP-dependent RNA helicase DHX30	2	1.04E-09	577803
Transcript_244563	mitochondrial import inner membrane translocase subunit TIM44	3	1.08E-09	752190
Transcript_438545	all-trans-retinol 13,14-reductase|putative all-trans-retinol 13,14-reductase	4	1.09E-09	581285
Transcript_172871	protein RER1	2	1.09E-09	579195
Transcript_417215	alanine--glyoxylate aminotransferase 2, mitochondrial	4	1.13E-09	594821

Transcript_256986	mitochondrial import inner membrane translocase subunit Tim10-B	3	1.16E-09	589835
Transcript_266569	stress-induced-phosphoprotein 1	3	1.17E-09	765087
Transcript_447903	uracil-DNA glycosylase	4	1.29E-09	586702
Transcript_342844	peroxiredoxin	2	1.31E-09	581408
Transcript_411315	propionyl-CoA carboxylase alpha chain, mitochondrial	2	1.40E-09	582365
Transcript_265130	RRP15-like protein	2	1.46E-09	577322
Transcript_358391	ribosome biogenesis protein NSA2 homolog	3	1.55E-09	577954
Transcript_264331	fibrillin-2|fibrillin-1	1	1.72E-09	582487
Transcript_455249	maleylacetoacetate isomerase	5	1.89E-09	580863
Transcript_443343	lysine--tRNA ligase	3	2.22E-09	588700
Transcript_393409	serine-enriched protein	2	2.28E-09	100888499
Transcript_264379	60 kDa heat shock protein, mitochondrial	2	2.33E-09	590510
Transcript_236885	serine/threonine-protein kinase rio2	2	2.44E-09	579218
Transcript_285991	hypoxia up-regulated protein 1|hypoxia up-regulated 1	3	2.63E-09	592339
Transcript_242646	60S ribosomal protein L8	2	2.67E-09	591341
Transcript_204616	T-complex protein 1 subunit delta	2	2.96E-09	579722
Transcript_194648	coiled-coil domain-containing protein 12	3	3.14E-09	585976
Transcript_410118	ATP-dependent RNA helicase DDX24	2	3.23E-09	578876
Transcript_232798	protein arginine N-methyltransferase 3	3	3.24E-09	588858
Transcript_243757	small nuclear ribonucleoprotein Sm D3	3	3.58E-09	105438529
Transcript_431275	eukaryotic translation initiation factor 2 subunit 2	3	3.61E-09	590481
Transcript_237829	gamma-butyrobetaine dioxygenase	1	3.73E-09	577198
Transcript_353055	deoxyhypusine hydroxylase	2	3.81E-09	582221
Transcript_351300	valine--tRNA ligase	3	3.92E-09	590476
Transcript_253045	cytochrome P450 10	2	4.01E-09	576441
Transcript_411364	CCR4-NOT transcription complex subunit 11	2	4.27E-09	594372
Transcript_215364	26S proteasome non-ATPase regulatory subunit 7	3	4.44E-09	575334
Transcript_78944	DNA-directed RNA polymerase I subunit RPA12	2	4.56E-09	100892475
Transcript_331128	deoxynucleotidyltransferase terminal-interacting protein 2	2	4.71E-09	586304
Transcript_285870	26S proteasome regulatory subunit 6A-B|26S protease regulatory subunit 6A-B|26S protease re	2	4.90E-09	576830
Transcript_324100	hydroxyacid oxidase 1	1	4.95E-09	584105
Transcript_280947	26S proteasome non-ATPase regulatory subunit 4	2	5.06E-09	575737
Transcript_279609	26S proteasome non-ATPase regulatory subunit 2	2	5.09E-09	588529
Transcript_367924	T-complex protein 1 subunit alpha	3	5.28E-09	582138
Transcript_359457	alpha-2-macroglobulin	3	5.54E-09	594798
Transcript_272476	leucine-rich repeat-containing protein 46	2	6.23E-09	100889057

Transcript_428533	proteasomal ubiquitin receptor ADRM1	2	6.25E-09	753175
Transcript_461744	serine--tRNA ligase, cytoplasmic	2	7.26E-09	588782
Transcript_437045	betaine--homocysteine S-methyltransferase 1	1	7.59E-09	752628
Transcript_218326	uncharacterized protein LOC100893287	1	7.60E-09	100893287
Transcript_420760	alkaline phosphatase	1	8.51E-09	580300
Transcript_454673	protein SDA1 homolog|SDA1 domain containing 1	3	8.75E-09	585590
Transcript_441945	queuosine salvage protein|UPF0553 protein C9orf64	2	8.75E-09	584962
Transcript_256633	bifunctional purine biosynthesis protein PURH	2	9.25E-09	588680
Transcript_386779	FRAS1-related extracellular matrix protein 1|FRAS1 related extracellular matrix 1	1	9.46E-09	591943
Transcript_297492	laminin subunit alpha-2|laminin subunit alpha-1	5	9.73E-09	587274
Transcript_397023	cadherin EGF LAG seven-pass G-type receptor 1	1	9.95E-09	105440441
Transcript_190981	superoxide dismutase [Cu-Zn]	1	1.02E-08	579361
Transcript_41462	EF-hand calcium-binding domain-containing protein 6	4	1.06E-08	589273
Transcript_271917	dihydroflavonol 4-reductase|putative NADPH-dependent methylglyoxal reductase GRP2|putati	3	1.09E-08	585152
Transcript_268427	steroid 17-alpha-hydroxylase/17,20 lyase	4	1.19E-08	585029
Transcript_384438	thyrotropin-releasing hormone-degrading ectoenzyme	1	1.20E-08	588093
Transcript_298313	spectrin alpha chain, non-erythrocytic 1	1	1.32E-08	580822
Transcript_437221	apoptosis-inducing factor 1, mitochondrial|apoptosis-inducing factor, mitochondrion-associated	3	1.37E-08	578253
Transcript_456290	claspin|claspin homolog	1	1.37E-08	575148
Transcript_343920	stAR-related lipid transfer protein 5	2	1.37E-08	584940
Transcript_293280	probable ribosome biogenesis protein RLP24	2	1.39E-08	581865
Transcript_245310	stAR-related lipid transfer protein 7, mitochondrial	2	1.42E-08	578525
Transcript_427274	contactin-2	1	1.44E-08	100893855
Transcript_431447	BRCA2 and CDKN1A-interacting protein	2	1.51E-08	586387
Transcript_461384	ATP-binding cassette sub-family C member 9	4	1.53E-08	594397
Transcript_404538	inactive C-alpha-formylglycine-generating enzyme 2	3	1.59E-08	576301
Transcript_185289	uncharacterized protein LOC100887836	1	1.75E-08	100887836
Transcript_381525	uronyl 2-sulfotransferase	1	1.76E-08	593546
Transcript_185057	GDH/6PGL endoplasmic bifunctional protein|hexose-6-phosphate dehydrogenase (glucose 1-de	1	2.02E-08	590387
Transcript_360124	cytochrome c	3	2.03E-08	575347
Transcript_295374	KH domain-containing, RNA-binding, signal transduction-associated protein 2	2	2.09E-08	588527
Transcript_386824	WD repeat-containing protein 74	2	2.16E-08	587044
Transcript_302605	proliferation-associated protein 2G4	2	2.23E-08	576641
Transcript_442775	Y+L amino acid transporter 2	2	2.24E-08	577249
Transcript_217735	protein RRP5 homolog|programmed cell death 11	3	2.75E-08	584784
Transcript_298943	LOW QUALITY PROTEIN: small nuclear ribonucleoprotein F	3	2.75E-08	592177

Transcript_276778	electron transfer flavoprotein beta subunit lysine methyltransferase|methyltransferase-like prc	3	2.88E-08	589785
Transcript_309454	microtubule-actin cross-linking factor 1|microtubule-actin cross-linking factor 1, isoforms 1/2/3	1	2.94E-08	756550
Transcript_395351	protein CDV3 homolog	2	3.00E-08	593799
Transcript_271655	RRP12-like protein	2	3.25E-08	587536
Transcript_444753	asparagine--tRNA ligase, cytoplasmic	2	3.27E-08	574833
Transcript_428848	26S proteasome non-ATPase regulatory subunit 6|proteasome (prosome, macropain) 26S subu	3	3.43E-08	590942
Transcript_431583	-	3	3.70E-08	100891280
Transcript_240340	tRNA methyltransferase 10 homolog A	3	3.72E-08	100889803
Transcript_199752	echinoidin-like	1	3.72E-08	105446729
Transcript_337159	ergothioneine biosynthesis protein 1|meiotically up-regulated gene 158 protein	1	4.12E-08	584365
Transcript_282269	dipeptidase 1	1	4.12E-08	592585
Transcript_393109	ran-specific GTPase-activating protein	2	4.20E-08	587798
Transcript_342318	aldehyde dehydrogenase, mitochondrial	1	4.23E-08	581706
Transcript_326720	U3 small nucleolar ribonucleoprotein protein IMP3	2	4.47E-08	590066
Transcript_214014	glutamate-rich WD repeat-containing protein 1	3	4.70E-08	581222
Transcript_240901	suppressor of SWI4 1 homolog	2	4.85E-08	582546
Transcript_306776	eukaryotic translation initiation factor 5B	3	5.25E-08	763128
Transcript_404542	uncharacterized protein LOC576204	4	5.64E-08	576204
Transcript_221851	deleted in malignant brain tumors 1 protein-like	1	5.78E-08	105440890
Transcript_322501	protein FAM199X	3	5.93E-08	100893786
Transcript_344436	replication termination factor 2|UPF0549 protein C20orf43 homolog|protein RTF2 homolog	2	5.99E-08	754268
Transcript_404038	39S ribosomal protein L19, mitochondrial	3	6.18E-08	589881
Transcript_283496	mitochondrial import inner membrane translocase subunit Tim23-like	3	6.32E-08	105440270
Transcript_376962	lambda-crystallin homolog	2	6.42E-08	589657
Transcript_265994	nascent polypeptide-associated complex subunit alpha	2	7.11E-08	576369
Transcript_311969	uncharacterized protein RAB5IF homolog|uncharacterized protein C20orf24 homolog	3	7.24E-08	576217
Transcript_340792	WD repeat-containing protein 82	3	7.30E-08	100888929
Transcript_219894	serine/arginine repetitive matrix protein 2	1	7.35E-08	100888995
Transcript_438464	FAD synthase	2	7.76E-08	593348
Transcript_311263	eukaryotic translation initiation factor 3 subunit M	2	7.76E-08	589955
Transcript_323033	tubulin beta chain	2	8.07E-08	373275
Transcript_359669	T-complex protein 1 subunit epsilon|chaperonin containing TCP1, subunit 5 (epsilon)	2	8.22E-08	575808
Transcript_215999	elongation factor Ts, mitochondrial	3	9.32E-08	578811
Transcript_430621	alkylglycerol monooxygenase	4	9.45E-08	579307
Transcript_229332	ADP-ribose pyrophosphatase, mitochondrial	4	1.04E-07	756760
Transcript_457631	zinc finger protein 330 homolog	2	1.05E-07	579935

Transcript_375389	N-terminal Xaa-Pro-Lys N-methyltransferase 1	3	1.06E-07	579562
Transcript_322851	nucleolar and coiled-body phosphoprotein 1	3	1.07E-07	591574
Transcript_225582	ADP-ribosylation factor	2	1.25E-07	763814
Transcript_205826	protein ABHD14B|abhydrolase domain-containing protein 14A-like|alpha/beta hydrolase doma	1	1.26E-07	585657
Transcript_448021	proteasome subunit alpha type-6	3	1.28E-07	589393
Transcript_434118	voltage dependent calcium channel L-type	1	1.32E-07	578128
Transcript_331800	uncharacterized protein LOC592324	2	1.33E-07	592324
Transcript_365923	insulin-like growth factor-binding protein complex acid labile subunit	1	1.33E-07	105447591
Transcript_403056	UTP15, U3 small nucleolar ribonucleoprotein, homolog (S. cerevisiae)-like	3	1.33E-07	754239
Transcript_205321	leucine-rich PPR motif-containing protein, mitochondrial	2	1.39E-07	755486
Transcript_291904	uncharacterized protein LOC105438010|Glycoside hydrolase family 31 domain containing prote	4	1.47E-07	105438010
Transcript_394693	folylpolyglutamate synthase, mitochondrial	2	1.49E-07	592205
Transcript_50290	uncharacterized protein LOC100890372	1	1.53E-07	100890372
Transcript_216838	LOW QUALITY PROTEIN: lipase maturation factor 2	4	1.57E-07	583944
Transcript_434160	ubiquitin-conjugating enzyme E2 N	3	1.57E-07	590968
Transcript_223216	uncharacterized protein LOC100892961	4	1.58E-07	100892961
Transcript_385327	amidophosphoribosyltransferase	2	1.71E-07	582049
Transcript_316114	eukaryotic translation initiation factor 2 subunit 3|eukaryotic translation initiation factor 2 subu	3	1.91E-07	582011
Transcript_364746	insulin-like growth factor 2 mRNA-binding protein 1	3	1.93E-07	576835
Transcript_334779	3'(2'),5'-bisphosphate nucleotidase 1	4	1.94E-07	580070
Transcript_185684	nucleolar protein 6	2	1.95E-07	577631
Transcript_196408	DNA mismatch repair protein Mlh3	2	1.97E-07	575007
Transcript_443664	acid phosphatase type 7|iron/zinc purple acid phosphatase-like protein	1	2.13E-07	580652
Transcript_429248	histone-binding protein RBBP4|retinoblastoma binding protein 4	3	2.13E-07	579451
Transcript_351461	28S ribosomal protein S22, mitochondrial	2	2.16E-07	589317
Transcript_454778	protein LLP homolog	2	2.26E-07	764101
Transcript_349228	uncharacterized protein LOC591826	2	2.30E-07	591826
Transcript_187299	transcription initiation protein SPT3 homolog	2	2.34E-07	586422
Transcript_361302	sideroflexin-5	1	2.64E-07	577642
Transcript_416648	nucleolar protein 14	3	2.78E-07	578764
Transcript_225955	endoribonuclease YbeY|putative ribonuclease|rRNA maturation factor homolog	1	2.82E-07	588773
Transcript_267958	regulator of chromosome condensation	3	2.90E-07	754700
Transcript_352669	zinc finger protein 593	2	2.96E-07	592435
Transcript_384255	zinc finger protein 362	2	2.99E-07	583226
Transcript_422610	hydroxyacid-oxoacid transhydrogenase, mitochondrial	1	3.05E-07	575433
Transcript_424059	acetyl-coenzyme A transporter 1	2	3.05E-07	589856

Transcript_332395	uncharacterized protein LOC591223	5	3.06E-07	591223
Transcript_225467	heat shock protein 75 kDa, mitochondrial	3	3.10E-07	578227
Transcript_444809	eukaryotic peptide chain release factor subunit 1	3	3.11E-07	591483
Transcript_195493	CTP synthase 1	2	3.11E-07	575088
Transcript_428926	menin	2	3.22E-07	589807
Transcript_197652	LOW QUALITY PROTEIN: tubulin alpha-1C chain	2	3.23E-07	762594
Transcript_203588	DDB1- and CUL4-associated factor 13	3	3.39E-07	589486
Transcript_280848	nudC domain-containing protein 1	2	3.39E-07	590657
Transcript_429226	uncharacterized protein LOC590843	4	3.46E-07	590843
Transcript_451457	PHD finger protein 24	3	3.49E-07	100892446
Transcript_343741	proteasome maturation protein	3	3.84E-07	579892
Transcript_185040	endothelin-converting enzyme 1	1	3.84E-07	755783
Transcript_282876	spermidine synthase	2	4.12E-07	591935
Transcript_427763	major facilitator superfamily domain-containing protein 9	2	4.19E-07	577847
Transcript_425248	T-complex protein 1 subunit gamma	3	4.26E-07	574601
Transcript_286893	proteasome subunit beta type-1	2	4.31E-07	754955
Transcript_323172	sialic acid synthase|N-acetylneuraminic acid synthase	2	4.43E-07	577884
Transcript_373212	fibrillin-1|latent-transforming growth factor beta-binding protein 4	1	4.48E-07	100887851
Transcript_177898	sodium- and chloride-dependent GABA transporter 1	1	4.60E-07	581958
Transcript_377252	neural-cadherin	1	4.62E-07	589984
Transcript_364897	FAST kinase domain-containing protein 4|protein TBRG4	3	4.73E-07	580380
Transcript_238012	G patch domain-containing protein 4|protein FAM133-like	3	4.75E-07	581552
Transcript_240840	uncharacterized protein LOC105439633|Autophagy-related protein 27	1	4.78E-07	105439633
Transcript_359750	glucosamine-6-phosphate isomerase 2	3	5.05E-07	585301
Transcript_309937	solute carrier organic anion transporter family member 2A1-like	2	5.28E-07	752367
Transcript_208822	lysophosphatidylserine lipase ABHD12|monoacylglycerol lipase ABHD12	2	5.35E-07	100891460
Transcript_418613	PIN2/TERF1-interacting telomerase inhibitor 1	2	5.37E-07	591382
Transcript_294332	60S ribosome subunit biogenesis protein NIP7 homolog	2	5.43E-07	579986
Transcript_353843	poly [ADP-ribose] polymerase tankyrase|poly(ADP-ribose) polymerase pme-5	4	5.86E-07	588883
Transcript_205105	uncharacterized protein LOC578177|low-density lipoprotein receptor-related protein 1B	1	5.99E-07	578177
Transcript_364601	acetolactate synthase-like protein	2	6.53E-07	593426
Transcript_321936	RNA-binding protein 34	3	6.77E-07	576551
Transcript_367437	secreted frizzled-related protein 3-like	3	6.84E-07	581781
Transcript_367877	vesicular integral-membrane protein VIP36|VIP36-like protein	3	7.04E-07	578461
Transcript_399833	serine/threonine-protein phosphatase CPPED1|calcineurin-like phosphoesterase domain-conta	2	7.07E-07	583499
Transcript_323748	cryptochrome-1	1	7.23E-07	580742

Transcript_394678	di-N-acetylchitobiase	2	7.28E-07	578451
Transcript_232547	protein LMBR1L	3	7.31E-07	578121
Transcript_340709	DNA polymerase beta	2	7.47E-07	582628
Transcript_393067	signal peptide, CUB and EGF-like domain-containing protein 1	1	8.42E-07	577317
Transcript_262552	cytochrome P450 3A9	1	8.45E-07	588156
Transcript_226152	myosin-16|myosin-6	1	8.72E-07	580674
Transcript_367772	tubulin alpha-1 chain	2	8.81E-07	105439767
Transcript_204519	mitochondrial-processing peptidase subunit alpha|peptidase (mitochondrial processing) alpha	3	9.15E-07	100889464
Transcript_6160	60S ribosomal protein L27a	2	9.97E-07	580148
Transcript_228286	elongation factor Tu, mitochondrial	3	1.02E-06	583958
Transcript_320536	4-hydroxyphenylpyruvate dioxygenase-like protein	3	1.03E-06	576414
Transcript_290528	TEF	3	1.06E-06	580363
Transcript_359106	GPI ethanolamine phosphate transferase 3	3	1.10E-06	584071
Transcript_302349	bystin	2	1.11E-06	578981
Transcript_260705	sorbin and SH3 domain-containing protein 1|vinexin	1	1.13E-06	574636
Transcript_205888	CDGSH iron-sulfur domain-containing protein 2 homolog A	1	1.13E-06	586672
Transcript_279040	KLF13	2	1.17E-06	576795
Transcript_230031	importin-7	2	1.18E-06	586884
Transcript_365373	ankyrin repeat and protein kinase domain-containing protein 1	3	1.24E-06	100889589
Transcript_278797	protein transport protein Sec24A|basic proline-rich protein-like	2	1.25E-06	579127
Transcript_305867	actin-5C	2	1.26E-06	581650
Transcript_303652	derlin-1	3	1.30E-06	592785
Transcript_344547	T-complex protein 1 subunit beta	2	1.33E-06	580864
Transcript_366717	uncharacterized protein LOC582810|Tudor domain-containing protein	2	1.33E-06	582810
Transcript_358088	fucose mutarotase	4	1.37E-06	100888310
Transcript_329212	eukaryotic translation initiation factor 3 subunit J	2	1.40E-06	578606
Transcript_344098	nucleolar GTP-binding protein 1|GTP binding protein 4	3	1.40E-06	575126
Transcript_374375	X-ray radiation resistance-associated protein 1	3	1.45E-06	584905
Transcript_198110	DNA-directed RNA polymerases I and III subunit RPAC1	3	1.45E-06	590827
Transcript_409226	CD151 antigen	4	1.46E-06	592232
Transcript_185863	interaptin|nuclear anchorage protein 1|nucleoprotein TPR|uncharacterized protein LOC58842	1	1.63E-06	588428
Transcript_411421	wnt inhibitory factor 1	1	1.70E-06	100892419
Transcript_393713	multifunctional protein ADE2	2	1.76E-06	575874
Transcript_212609	NHL repeat-containing protein 2	2	1.83E-06	591178
Transcript_441990	uncharacterized protein LOC100888048	1	1.85E-06	100888048
Transcript_243213	protein-serine O-palmitoleoyltransferase porcupine|protein-cysteine N-palmitoyltransferase po	2	1.98E-06	577959

Transcript_249746	DNA-directed RNA polymerase, mitochondrial	2	1.98E-06	762772
Transcript_286756	GDP-fucose protein O-fucosyltransferase 1	3	2.03E-06	585387
Transcript_235731	MAM and LDL-receptor class A domain-containing protein 2	1	2.05E-06	755161
Transcript_440018	nucleolar complex protein 4 homolog	2	2.11E-06	591488
Transcript_341101	A disintegrin and metalloproteinase with thrombospondin motifs 18|ADAM metallopeptidase w	3	2.36E-06	574964
Transcript_180918	phosphopantothenoylcysteine decarboxylase subunit SIS2-like	1	2.38E-06	105438224
Transcript_439703	protein CBFA2T1|protein CBFA2T3-like	1	2.40E-06	576535
Transcript_405538	uncharacterized protein LOC100891656	1	2.45E-06	100891656
Transcript_292194	FERM domain-containing protein 1	1	2.46E-06	592633
Transcript_110850	60S ribosomal protein L3	2	2.89E-06	586477
Transcript_434866	uncharacterized protein LOC589939|Harbinger transposase-derived nuclease domain-containir	3	2.92E-06	589939
Transcript_438152	DNA excision repair protein ERCC-6-like|transcriptional regulator ATRX	1	2.95E-06	575953
Transcript_446005	2-oxoglutarate-dependent dioxygenase htyE|UPF0676 protein C1494.01	1	3.10E-06	752472
Transcript_286899	uncharacterized protein LOC587999	2	3.21E-06	587999
Transcript_325335	uncharacterized protein LOC593876	1	3.21E-06	593876
Transcript_249330	eukaryotic translation initiation factor 3 subunit H-B	2	3.27E-06	575080
Transcript_443947	uncharacterized protein LOC579607|putative oxidoreductase YteT-like	1	3.33E-06	579607
Transcript_190658	pyrroline-5-carboxylate reductase 3	2	3.41E-06	586502
Transcript_443207	Bardet-Biedl syndrome 1 protein	2	3.41E-06	578507
Transcript_350598	RNA 3'-terminal phosphate cyclase-like protein	2	3.67E-06	592597
Transcript_239281	cholinesterase 1|thyroglobulin	1	3.85E-06	583460
Transcript_367596	transitional endoplasmic reticulum ATPase	3	3.99E-06	575609
Transcript_381250	prostaglandin E synthase 2	3	4.04E-06	584887
Transcript_409717	isochorismatase domain-containing protein 2|isochorismatase domain-containing protein 2, mi	4	4.04E-06	590365
Transcript_185238	multifunctional methyltransferase subunit TRM112-like protein|tRNA methyltransferase 112 ho	2	4.21E-06	579707
Transcript_444350	melanotransferrin	3	4.37E-06	581063
Transcript_306787	methyltransferase-like protein 24	1	4.40E-06	100891683
Transcript_271897	HEAT repeat-containing protein 1	3	4.60E-06	589888
Transcript_302913	L-lactate dehydrogenase	1	4.65E-06	586683
Transcript_386663	pancreatic lipase-related protein 2	1	4.67E-06	592851
Transcript_432931	NADPH oxidase 5	1	4.74E-06	752031
Transcript_306891	graves disease carrier protein homolog	2	4.80E-06	584753
Transcript_382713	60S ribosomal protein L19	2	4.80E-06	593861
Transcript_416522	uncharacterized protein LOC100888035	2	4.85E-06	100888035
Transcript_431677	coatomer subunit beta'	2	4.95E-06	589321
Transcript_185030	DNA-dependent protein kinase catalytic subunit	1	5.16E-06	586799

Transcript_381229	Golgi SNAP receptor complex member 1	2	5.23E-06	593134
Transcript_301702	unconventional myosin-XVI|formin-like protein 20|myosin XVI|putative F-box protein At1g473	1	5.29E-06	587143
Transcript_197661	LOW QUALITY PROTEIN: tubulin alpha-1 chain	2	5.35E-06	588319
Transcript_206262	cleavage stimulation factor subunit 2	2	5.44E-06	753333
Transcript_316435	neprilysin-1|endothelin-converting enzyme 1	1	5.66E-06	580325
Transcript_266841	tuftelin	4	5.71E-06	576303
Transcript_319974	uncharacterized protein LOC100890537	1	5.79E-06	100890537
Transcript_223102	transducin beta-like protein 3	3	6.10E-06	100891641
Transcript_234991	ADAMTS-like protein 1	2	6.11E-06	580296
Transcript_277138	uncharacterized protein LOC100891149	3	6.28E-06	100891149
Transcript_43695	b(0,+)-type amino acid transporter 1|B(0,+)-type amino acid transporter 1-like	1	6.33E-06	579553
Transcript_447128	maltase 1|alpha-glucosidase	1	6.40E-06	583103
Transcript_190954	uncharacterized protein LOC593642|CAP domain-containing protein	4	6.55E-06	593642
Transcript_84237	actin CyI, cytoplasmic	1	6.60E-06	592912
Transcript_412252	protein SEC13 homolog	3	6.76E-06	583776
Transcript_284067	programmed cell death protein 2-like	2	6.81E-06	576484
Transcript_262309	N-alpha-acetyltransferase 11	2	6.82E-06	580091
Transcript_396324	phosphopantothenate--cysteine ligase	3	6.92E-06	763180
Transcript_302120	cadherin-23|fat-like cadherin-related tumor suppressor homolog|protocadherin Fat 1	1	6.98E-06	594286
Transcript_433507	MKX	2	7.15E-06	105436421
Transcript_205651	lysophosphatidylcholine acyltransferase 2	1	7.37E-06	579107
Transcript_202496	ribosome biogenesis protein BRX1 homolog	3	7.55E-06	583114
Transcript_425468	dnaJ homolog subfamily B member 1	2	7.82E-06	752244
Transcript_315415	putative uncharacterized protein CXorf58	2	7.85E-06	593302
Transcript_244512	aspartate--tRNA ligase, cytoplasmic	3	8.02E-06	588558
Transcript_272010	proto-oncogene Wnt-3	3	8.05E-06	585683
Transcript_285582	splicing factor 3A subunit 2	2	8.28E-06	576257
Transcript_438398	U2 small nuclear ribonucleoprotein B''|U1 small nuclear ribonucleoprotein A-like	3	8.35E-06	584276
Transcript_289715	uncharacterized protein LOC763307|ZU5 domain-containing protein	3	8.56E-06	763307
Transcript_328267	40S ribosomal protein S26	2	8.78E-06	576654
Transcript_356949	ubiquitin-conjugating enzyme E2 K	2	9.01E-06	585095
Transcript_440889	serine/threonine-protein phosphatase 4 catalytic subunit	2	9.06E-06	594644
Transcript_405218	40S ribosomal protein S11	2	9.09E-06	579026
Transcript_375963	uncharacterized protein LOC581850	1	9.12E-06	581850
Transcript_299201	mesencephalic astrocyte-derived neurotrophic factor homolog	3	9.30E-06	755636
Transcript_215281	decaprenyl-diphosphate synthase subunit 2	2	9.56E-06	580439

Transcript_368597	ubiquitin carboxyl-terminal hydrolase 14	2	1.00E-05	581896
Transcript_346637	nuclear migration protein nudC	2	1.01E-05	589717
Transcript_351605	26S proteasome non-ATPase regulatory subunit 10	3	1.02E-05	576864
Transcript_244261	acidic leucine-rich nuclear phosphoprotein 32 family member E	2	1.02E-05	100892466
Transcript_440816	tyrosine aminotransferase	2	1.02E-05	592114
Transcript_381701	rho-related BTB domain-containing protein 1	1	1.02E-05	575159
Transcript_445237	39S ribosomal protein S30, mitochondrial|28S ribosomal protein S30, mitochondrial	3	1.06E-05	591808
Transcript_298879	DNA-directed RNA polymerase III subunit RPC6	2	1.08E-05	580860
Transcript_275685	uncharacterized protein LOC587541	4	1.09E-05	587541
Transcript_381489	papilin	1	1.12E-05	584177
Transcript_362661	ribosome-recycling factor, mitochondrial	3	1.13E-05	587941
Transcript_179642	tetraspanin-5	1	1.14E-05	582219
Transcript_404610	uncharacterized protein F13E9.13, mitochondrial|Membrane complex biogenesis protein, BtpA	3	1.14E-05	579795
Transcript_310943	tryptophan--tRNA ligase, cytoplasmic	2	1.16E-05	574927
Transcript_180218	proton-coupled amino acid transporter 1	4	1.20E-05	587558
Transcript_277419	protein SET	2	1.23E-05	583082
Transcript_316855	deleted in malignant brain tumors 1 protein|galectin-3-binding protein pseudogene|scavenger	1	1.25E-05	754160
Transcript_217175	mitochondrial import inner membrane translocase subunit Tim22	3	1.27E-05	592058
Transcript_271076	carbohydrate sulfotransferase 15	4	1.34E-05	582046
Transcript_434835	alpha-aminoadipic semialdehyde dehydrogenase	1	1.34E-05	588376
Transcript_412907	tubulin alpha-2/alpha-4 chain	2	1.35E-05	582621
Transcript_429645	N-acetylated-alpha-linked acidic dipeptidase 2-like	1	1.36E-05	583418
Transcript_235859	sucrase-isomaltase, intestinal	1	1.40E-05	588081
Transcript_267184	28S ribosomal protein S5, mitochondrial	3	1.43E-05	583862
Transcript_380459	cryptochrome-2	4	1.46E-05	583959
Transcript_453750	uncharacterized protein LOC581715|probable enoyl-CoA hydratase echA8-like|probable enoyl-	4	1.49E-05	581715
Transcript_213782	nicalin-1	3	1.50E-05	587749
Transcript_323832	aminoacylase-1	1	1.70E-05	577570
Transcript_402336	mitochondrial ribonuclease P catalytic subunit|mitochondrial ribonuclease P protein 3	3	1.71E-05	578934
Transcript_359571	LOW QUALITY PROTEIN: uncharacterized protein LOC577952	1	1.71E-05	577952
Transcript_189718	MYBBP1A	2	1.71E-05	583345
Transcript_388247	mitochondrial glutamate carrier 2	2	1.74E-05	753367
Transcript_369099	protein Abitram|protein FAM206A-like|protein Simiate	3	1.75E-05	757335
Transcript_305359	metabotropic glutamate receptor 1|extracellular calcium-sensing receptor-like	3	1.75E-05	577623
Transcript_456418	ficolin-2-like	1	1.80E-05	583514
Transcript_433359	probable serine/threonine-protein kinase drkD|putative serine/threonine-protein kinase drkD	2	1.85E-05	577165

Transcript_360852	ribosomal RNA-processing protein 7 homolog A	3	1.88E-05	580493
Transcript_236563	protein FAM166B-like	5	1.92E-05	105439658
Transcript_322912	39S ribosomal protein L13, mitochondrial	3	2.04E-05	585375
Transcript_373532	putative helicase MOV-10	3	2.05E-05	583129
Transcript_374213	uncharacterized protein LOC763830	3	2.06E-05	763830
Transcript_442862	zinc finger CCCH domain-containing protein 15	2	2.07E-05	580460
Transcript_401581	protein canopy homolog 2	3	2.08E-05	588644
Transcript_453440	glutamate receptor 2	1	2.10E-05	591929
Transcript_343792	elongation factor 1-delta-like	3	2.16E-05	575168
Transcript_371946	uncharacterized protein LOC100889820	4	2.23E-05	100889820
Transcript_337768	coiled-coil domain-containing protein 58	3	2.23E-05	576818
Transcript_230454	exportin-5	3	2.31E-05	592021
Transcript_314891	periodic tryptophan protein 2 homolog	3	2.43E-05	752047
Transcript_321961	organic cation transporter protein	1	2.46E-05	582688
Transcript_299268	39S ribosomal protein L52, mitochondrial	3	2.49E-05	752505
Transcript_227249	cytochrome P450 2J6	1	2.49E-05	575697
Transcript_329073	uncharacterized protein LOC100889633	2	2.50E-05	100889633
Transcript_449172	transcription initiation factor IIA subunit 1	2	2.52E-05	100888038
Transcript_66007	cytospin-A	1	2.56E-05	583396
Transcript_423340	inverted formin-2	1	2.58E-05	579910
Transcript_423272	phe13-bombesin receptor	1	2.75E-05	100888815
Transcript_276375	uncharacterized protein LOC584281	1	2.75E-05	584281
Transcript_243879	carbonyl reductase [NADPH] 1	4	2.76E-05	574551
Transcript_310622	actin related protein 1	1	2.83E-05	373192
Transcript_380140	cardiolipin synthase (CMP-forming)|probable cardiolipin synthase (CMP-forming)	2	2.86E-05	587370
Transcript_378334	DNA-directed RNA polymerase II subunit RPB7	3	2.88E-05	752151
Transcript_298921	development-specific protein LVN1.2	1	2.90E-05	100888941
Transcript_393283	LOW QUALITY PROTEIN: xanthine dehydrogenase/oxidase	1	3.15E-05	576712
Transcript_388184	serine/arginine repetitive matrix protein 1	1	3.16E-05	575766
Transcript_401245	uncharacterized protein LOC593760	4	3.16E-05	593760
Transcript_434004	hydroxyacyl-coenzyme A dehydrogenase, mitochondrial	3	3.18E-05	582126
Transcript_384548	salivary glue protein Sgs-3	3	3.18E-05	100892638
Transcript_380262	cytochrome P450 4V2	4	3.20E-05	581875
Transcript_42693	40S ribosomal protein S13	2	3.25E-05	581196
Transcript_206502	nucleolar protein 56|NOP56 ribonucleoprotein homolog	2	3.29E-05	590067
Transcript_195549	sodium-dependent phosphate transporter 2	2	3.35E-05	582615

Transcript_331211	probable Na(+)/H(+) antiporter nhx-9	1	3.38E-05	591586
Transcript_372883	protein dispatched homolog 1-like	1	3.39E-05	580689
Transcript_308094	sodium-dependent dopamine transporter|solute carrier family 6 (neurotransmitter transporter	1	3.48E-05	584179
Transcript_438519	degenerin deg-1	1	3.53E-05	100891272
Transcript_307696	ATP-binding cassette sub-family B member 8, mitochondrial	3	3.69E-05	754756
Transcript_370380	serine/threonine-protein kinase NLK|serine/threonine-protein kinase NLK2	2	3.73E-05	574783
Transcript_402303	GTP 3',8-cyclase, mitochondrial|cyclic pyranopterin monophosphate synthase, mitochondrial|n	4	3.77E-05	578700
Transcript_340758	proteasome subunit alpha type-2	3	3.77E-05	579111
Transcript_423204	FOXA	1	3.80E-05	578584
Transcript_443748	pyridine nucleotide-disulfide oxidoreductase domain-containing protein 2|pyridine nucleotide-[illegible]	4	3.86E-05	587519
Transcript_426155	mitotic apparatus protein p62	3	3.98E-05	105443132
Transcript_373050	dihydropteridine reductase	1	4.06E-05	575893
Transcript_268252	39S ribosomal protein L47, mitochondrial	3	4.15E-05	588415
Transcript_261599	elongation factor 1 alpha	2	4.40E-05	548620
Transcript_315154	peroxisomal membrane protein 11A	1	4.41E-05	576910
Transcript_254082	probable ATP-dependent RNA helicase DDX56	2	4.51E-05	585968
Transcript_394798	band 7 protein AGAP004871-like	1	4.55E-05	578433
Transcript_253869	uncharacterized protein LOC100889419|IgGFc-binding protein, N-terminal domain containing p	1	4.65E-05	100889419
Transcript_258821	transcription initiation factor TFIID subunit 10	2	4.67E-05	752563
Transcript_433800	proteasome subunit alpha type-7	2	4.80E-05	579458
Transcript_459696	MYNN	2	4.88E-05	100893972
Transcript_347359	ectonucleotide pyrophosphatase/phosphodiesterase family member 7-like	5	4.90E-05	577518
Transcript_355848	NACHT domain- and WD repeat-containing protein 1|NACHT and WD repeat domain-containing	1	4.97E-05	591208
Transcript_196171	peptidyl-prolyl cis-trans isomerase	2	5.02E-05	585216
Transcript_139365	deleted in malignant brain tumors 1 protein	1	5.10E-05	753862
Transcript_267269	39S ribosomal protein L23, mitochondrial	2	5.29E-05	587414
Transcript_219950	citrate synthase, mitochondrial	2	5.60E-05	590444
Transcript_223195	15-hydroxyprostaglandin dehydrogenase [NAD(+)]	4	5.63E-05	591898
Transcript_230867	RNA N6-adenosine-methyltransferase mettl16|methyltransferase-like protein 16	3	5.67E-05	588854
Transcript_279744	eukaryotic translation initiation factor 4 gamma 3|eukaryotic translation initiation factor 4 gam	2	5.68E-05	588274
Transcript_330100	NRL/MAF	1	5.85E-05	581716
Transcript_254029	uncharacterized protein LOC105441448	1	6.12E-05	105441448
Transcript_431131	proteasome subunit beta type-7	3	6.38E-05	588252
Transcript_263253	E3 ubiquitin-protein ligase TRIM23	1	6.38E-05	591350
Transcript_364264	DNA polymerase epsilon subunit 4	2	6.53E-05	763808
Transcript_181686	patched domain-containing protein 3	2	6.55E-05	100888465

Transcript_203172	uncharacterized protein LOC577523|malate dehydrogenase-like	1	6.68E-05	577523
Transcript_427147	carbohydrate sulfotransferase 11	1	6.68E-05	589022
Transcript_414827	guanine nucleotide exchange factor MSS4	2	6.83E-05	591831
Transcript_197695	quinone oxidoreductase-like|uncharacterized protein LOC764164|Alcohol dehydrogenase, C-te	2	7.08E-05	764164
Transcript_269277	ribosomal protein S14	2	7.19E-05	574895
Transcript_246683	PMS1 protein homolog 1	1	7.21E-05	592483
Transcript_447058	coiled-coil domain-containing protein 86|uncharacterized protein LOC100889012	3	7.35E-05	100889012
Transcript_428449	COX assembly mitochondrial protein homolog	3	7.46E-05	754298
Transcript_373487	leucine-rich repeat-containing protein 58	2	7.64E-05	592464
Transcript_297301	isthmin	2	8.01E-05	762678
Transcript_274172	60S ribosomal protein L31	2	8.02E-05	589085
Transcript_214012	RNA transcription, translation and transport factor protein|UPF0568 protein C14orf166 homolo	3	8.11E-05	586176
Transcript_180063	endothelin-converting enzyme homolog	1	8.20E-05	594281
Transcript_335203	cyclin-dependent kinase 2	1	8.26E-05	585950
Transcript_436916	scavenger receptor cysteine-rich domain-containing group B protein	1	8.28E-05	577634
Transcript_168671	EEF1A1	2	8.38E-05	548620
Transcript_196792	hydroxyproline dehydrogenase	1	8.47E-05	576696
Transcript_437063	serine/threonine-protein kinase TBK1	3	8.56E-05	579073
Transcript_269134	octopamine receptor	2	8.60E-05	100893874
Transcript_280627	methylenetetrahydrofolate reductase	1	8.96E-05	589576
Transcript_317810	kin of IRRE-like protein 1	1	9.04E-05	762504
Transcript_387048	peptidyl-prolyl cis-trans isomerase-like 1	2	9.09E-05	575873
Transcript_316602	DNA mismatch repair protein Msh2|mutS homolog 2, colon cancer, nonpolyposis type 1	1	9.13E-05	589363
Transcript_263803	phosphate carrier protein, mitochondrial	2	9.29E-05	577743
Transcript_249042	thioredoxin-like protein 1	3	9.29E-05	581305
Transcript_323338	probable assembly chaperone of rpl4|UPF0661 TPR repeat-containing protein C16D10.01c	2	9.32E-05	100891594
Transcript_218894	40S ribosomal protein S15a|ribosomal protein S24	2	9.49E-05	373469
Transcript_77032	cubilin	2	9.56E-05	580195
Transcript_214740	glycerophosphocholine phosphodiesterase GPCPD1	1	9.94E-05	594641
Transcript_381580	short-chain collagen C4	1	9.95E-05	100893425
Transcript_373705	tctex1 domain-containing protein 1	1	0.000100228	588988
Transcript_255002	protein giant	2	0.000100627	105444027
Transcript_328974	GRAM domain-containing protein 4	1	0.00010098	581215
Transcript_294422	persulfide dioxygenase ETHE1, mitochondrial	5	0.000102325	585574
Transcript_203149	DNA-directed RNA polymerases I, II, and III subunit RPABC1	3	0.00010449	578317
Transcript_360539	methionine adenosyltransferase	3	0.00010822	548618

Transcript_73952	aminoacyl tRNA synthase complex-interacting multifunctional protein 2	2	0.00010822	575132
Transcript_303046	U3 small nucleolar RNA-associated protein 14 homolog A	3	0.000111727	585850
Transcript_199821	probable ATP-dependent RNA helicase DDX47	3	0.000112486	581057
Transcript_175282	uncharacterized protein LOC105442054	4	0.000113657	105442054
Transcript_267731	39S ribosomal protein L12, mitochondrial	3	0.000120962	585458
Transcript_461577	deoxyribodipyrimidine photo-lyase	1	0.000123123	593941
Transcript_448396	receptor for egg jelly protein	1	0.000127512	576647
Transcript_88669	40S ribosomal protein S5	2	0.000129515	592668
Transcript_383623	histidine ammonia-lyase	1	0.000131478	583726
Transcript_247937	EGR1	1	0.000141967	100892559
Transcript_368914	protein CNPPD1	2	0.000154939	752566
Transcript_428975	RING finger protein 215	4	0.000155181	753231
Transcript_235182	programmed cell death protein 2	3	0.000155446	585086
Transcript_280475	major facilitator superfamily domain-containing protein 6	3	0.000157081	763022
Transcript_459174	notchless protein homolog 1	2	0.000161391	585438
Transcript_411220	RING-box protein 1	3	0.000167486	591601
Transcript_88201	40S ribosomal protein S4	2	0.000169885	583495
Transcript_339559	lysozyme 3	1	0.000170656	583337
Transcript_195407	xylulose kinase|xylulokinase homolog (H. influenzae)	1	0.000170904	589304
Transcript_265718	sodium-dependent phosphate transport protein 2C	2	0.000171783	576137
Transcript_253116	dynein regulatory complex subunit 6|F-box/LRR-repeat protein 13	1	0.000175152	577951
Transcript_448836	tubulointerstitial nephritis antigen-like	1	0.000176097	576823
Transcript_215764	U6 snRNA-associated Sm-like protein LSm2	3	0.000186826	587779
Transcript_443991	oxidoreductase HTATIP2-like	3	0.000191678	592505
Transcript_276623	histone H4	2	0.000196637	752213
Transcript_360643	nucleoside diphosphate kinase B	3	0.000199818	594617
Transcript_345101	serine/threonine-protein kinase Nek6	1	0.00020157	100893147
Transcript_445434	28S ribosomal protein S17, mitochondrial	3	0.000201605	753889
Transcript_352645	arginine--tRNA ligase, cytoplasmic	3	0.000202136	575790
Transcript_199943	lens fiber major intrinsic protein	1	0.000202136	594743
Transcript_253163	venom phosphodiesterase 2	1	0.000202136	579858
Transcript_278701	NHP2-like protein 1	3	0.000206072	587294
Transcript_220262	uncharacterized protein LOC581634	2	0.000206087	581634
Transcript_314700	uncharacterized protein LOC587761	1	0.000207742	587761
Transcript_402975	eukaryotic initiation factor 4A-III	2	0.000209348	580266
Transcript_284772	TAF1A	2	0.000213915	105440467

Transcript_361334	chordin	1	0.000215196	580173
Transcript_457700	lengsin-like	2	0.000216409	591380
Transcript_300296	trimeric intracellular cation channel type 1B.1|trimeric intracellular cation channel type A	4	0.000227596	100890448
Transcript_338151	histone H3, embryonic	2	0.000227596	581030
Transcript_383403	mediator of RNA polymerase II transcription subunit 18	3	0.000232301	576519
Transcript_456392	RIIa domain-containing protein 1	3	0.00023765	752632
Transcript_299206	39S ribosomal protein L49, mitochondrial	2	0.000241306	593029
Transcript_383531	natural resistance-associated macrophage protein 2	2	0.000251469	576440
Transcript_327352	macrophage mannose receptor 1	1	0.000256987	100893009
Transcript_172390	protein SSUH2 homolog	1	0.000257132	763611
Transcript_353965	uncharacterized protein LOC100892584	2	0.0002581	100892584
Transcript_454078	RNA-binding protein NOB1	2	0.000262544	575793
Transcript_443183	MYC	2	0.000262997	373385
Transcript_329842	SRA stem-loop-interacting RNA-binding protein, mitochondrial-like	3	0.000265293	754241
Transcript_464404	peroxiredoxin-1	2	0.000271849	590165
Transcript_380053	cholinesterase 1	1	0.000275228	591159
Transcript_462380	metalloproteinase inhibitor 3	3	0.000279283	100889149
Transcript_372078	potassium voltage-gated channel subfamily KQT member 4|potassium voltage-gated channel su	3	0.000280671	579971
Transcript_206791	uncharacterized protein LOC105442064|Death effector domain-containing protein	1	0.000288649	105442064
Transcript_86447	glyceraldehyde-3-phosphate dehydrogenase	2	0.000304165	578260
Transcript_392219	neuropeptide Y receptor type 2-like	3	0.000306609	100891742
Transcript_327467	chromosome transmission fidelity protein 8 homolog	3	0.000309435	763690
Transcript_308925	dnaJ homolog subfamily C member 2	2	0.000310386	583525
Transcript_202368	eukaryotic initiation factor 4a	2	0.000311754	575736
Transcript_166464	translation elongation factor 2	2	0.000315605	592801
Transcript_179487	uncharacterized protein LOC580673	4	0.000318961	580673
Transcript_134695	angiotensin-converting enzyme 2	1	0.000330507	580220
Transcript_280569	TNF receptor-associated factor 6-A	3	0.000332355	585060
Transcript_255311	WD repeat-containing protein 37	1	0.000344097	581583
Transcript_461472	growth hormone secretagogue receptor type 1|neuropeptides capa receptor|growth hormone	2	0.000351928	592924
Transcript_345624	40S ribosomal protein S9	2	0.000351928	586110
Transcript_230874	ubiquitin	2	0.000355053	584839
Transcript_25798	ATP synthase F(0) complex subunit C2, mitochondrial|ATP synthase lipid-binding protein, mitoc	3	0.000360871	583818
Transcript_397056	coatomer subunit epsilon	3	0.000367007	582090
Transcript_333839	G2/mitotic-specific cyclin-B3	5	0.000367007	591240
Transcript_416611	zinc transporter ZIP12	1	0.000368191	587958

Transcript_196818	UDP-glucuronosyltransferase 2C1|UDP-glucuronosyltransferase 1-2|UDP-glucuronosyltransfera	1	0.000369716	589187
Transcript_280665	uncharacterized protein LOC100890259	1	0.000376789	100890259
Transcript_249751	putative Dol-P-Glc:Glc(2)Man(9)GlcNAc(2)-PP-Dol alpha-1,2-glucosyltransferase	3	0.000383409	590617
Transcript_483697	ras-related protein ORAB-1	2	0.000384483	373418
Transcript_323044	pleiotropic regulator 1	2	0.000388456	756406
Transcript_189980	uncharacterized protein LOC100890349|L-idonate 5-dehydrogenase	1	0.000406171	100890349
Transcript_301658	nucleolar protein 10	2	0.000408603	590723
Transcript_459372	fibrillin-3|bypass of stop codon protein 1	4	0.000410913	591161
Transcript_199327	delta-aminolevulinic acid dehydratase	2	0.00041335	577020
Transcript_183069	glutamate receptor 1	2	0.000415045	587837
Transcript_192833	5-oxoprolinase|5-oxoprolinase (ATP-hydrolysing)	1	0.000427737	589767
Transcript_373239	ras-related protein Rap-2c	2	0.000440545	753090
Transcript_386406	WD repeat-containing protein 43	2	0.000451267	592550
Transcript_238124	protein MAK16 homolog A	2	0.000460165	586606
Transcript_233725	uncharacterized protein C2orf50	1	0.000494308	578348
Transcript_300378	CDP-diacylglycerol--glycerol-3-phosphate 3-phosphatidyltransferase, mitochondrial	3	0.000520562	581340
Transcript_421581	pre-rRNA-processing protein TSR2 homolog	2	0.000525637	592405
Transcript_236174	40S ribosomal protein S3	2	0.000532436	593744
Transcript_249550	sorbitol dehydrogenase	1	0.000534208	585570
Transcript_234548	angiopoietin-4-like	1	0.0005769	100891514
Transcript_128686	pre-rRNA 2'-O-ribose RNA methyltransferase FTSJ3|pre-rRNA processing protein FTSJ3	3	0.000590448	582089
Transcript_333875	uncharacterized protein LOC578017	5	0.000597208	578017
Transcript_208042	ST8 alpha-N-acetyl-neuraminide alpha-2,8-sialyltransferase 4	1	0.000631501	586329
Transcript_257273	methylsterol monooxygenase 1	1	0.000641983	100891893
Transcript_308851	alcohol dehydrogenase class-3	1	0.000649203	579220
Transcript_385086	pre-piRNA 3'-exonuclease trimmer|poly(A)-specific ribonuclease PARN-like domain-containing	2	0.000665071	100891016
Transcript_408947	phosphopantothenoylcysteine decarboxylase	3	0.000674726	584017
Transcript_273618	SHC SH2 domain-binding protein 1	4	0.000680453	575406
Transcript_418431	ketimine reductase mu-crystallin	1	0.000684468	579068
Transcript_214978	basic leucine zipper and W2 domain-containing protein 1	2	0.000692153	587479
Transcript_432419	beta-lactamase domain-containing protein 2	1	0.000718546	575771
Transcript_196327	creatine kinase, flagellar	5	0.00071996	580751
Transcript_221788	NADPH oxidase	1	0.000721382	757329
Transcript_329183	E3 ubiquitin-protein ligase UHRF1|histone-lysine N-methyltransferase, H3 lysine-9 specific SUV	1	0.000725484	756912
Transcript_346536	cAMP-dependent protein kinase catalytic subunit 1|catalytic subunit of cAMP-dependent histor	2	0.000751818	589947
Transcript_366071	N-acetylgalactosamine-6-sulfatase-like	1	0.000766197	579130

Transcript_235911	phospholipase B1, membrane-associated	1	0.000775871	100890912
Transcript_403482	aldo-keto reductase family 1 member A1|alcohol dehydrogenase	4	0.000784233	576680
Transcript_334842	uncharacterized protein LOC580196	2	0.000786461	580196
Transcript_235072	uncharacterized protein LOC579112	4	0.000788949	579112
Transcript_82280	calmodulin	2	0.000790274	575365
Transcript_424005	soma ferritin	3	0.000806	591499
Transcript_419016	dnaJ homolog subfamily C member 5|cysteine string protein	2	0.000807136	577697
Transcript_12298	40S ribosomal protein S23	2	0.000841834	590530
Transcript_368000	syntenin-1	2	0.000847174	578747
Transcript_448298	protein Wnt-7b	1	0.000850909	581981
Transcript_374203	protein Asterix	3	0.000866756	592477
Transcript_121057	V-type proton ATPase 16 kDa proteolipid subunit	2	0.000903696	593221
Transcript_276903	ERI1 exoribonuclease 2	1	0.00096432	585927
Transcript_388827	ADP/ATP translocase 3	2	0.000969427	575225
Transcript_240937	uracil phosphoribosyltransferase homolog	2	0.000983598	575128
Transcript_444850	probable RNA-binding protein EIF1AD|putative RNA-binding protein EIF1AD	3	0.000991161	578771
Transcript_427937	DNA repair protein XRCC3	3	0.00101429	763443
Transcript_286704	extended synaptotagmin-2	5	0.001044619	579674
Transcript_263515	polycystic kidney disease protein 1-like 2	1	0.001052967	579144
Transcript_357605	glutathione S-transferase P	3	0.001055612	580662
Transcript_438353	EMX1	4	0.001088813	577702
Transcript_264220	ADP-ribosylation factor 1|ADP-ribosylation factor-like	3	0.001110619	586151
Transcript_208170	uncharacterized protein LOC105441928	1	0.001110863	105441928
Transcript_383458	probable 60S ribosomal protein L37-A	2	0.001114113	591582
Transcript_51640	RCC1 and BTB domain-containing protein 1|ultraviolet-B receptor UVR8|probable E3 ubiquitin-	4	0.001129851	577290
Transcript_305797	WD repeat-containing protein 31	1	0.001163089	585021
Transcript_240632	calpain small subunit 1	4	0.001196235	100889224
Transcript_171991	protein cornichon homolog 1	3	0.00119957	582532
Transcript_398758	serine/threonine-protein phosphatase PGAM5, mitochondrial	2	0.001214001	588383
Transcript_353555	caspase-6	1	0.001318326	584221
Transcript_188732	tetratricopeptide repeat protein 32	2	0.001350098	588488
Transcript_264574	conserved oligomeric Golgi complex subunit 2|component of oligomeric golgi complex 2	3	0.001377664	589392
Transcript_392565	methionyl-tRNA formyltransferase, mitochondrial	2	0.001379209	586344
Transcript_235778	ST8 alpha-N-acetyl-neuraminide alpha-2,8-sialyltransferase 7|TM2 domain-containing protein	1	0.001456409	583563
Transcript_463187	mitochondrial import inner membrane translocase subunit Tim10 B	2	0.001462773	574660
Transcript_359454	dynein light chain LC6, flagellar outer arm	4	0.001472937	591043

Transcript_402965	PRELI domain containing protein 3B|protein slowmo homolog 2	2	0.001481224	100889391
Transcript_434653	pre-rRNA-processing protein TSR1 homolog|TSR1, 20S rRNA accumulation, homolog	2	0.001486958	594000
Transcript_323407	uncharacterized protein LOC763845	1	0.001498958	763845
Transcript_456790	acyloxyacyl hydrolase	1	0.001508263	592185
Transcript_309275	uncharacterized protein LOC100889927	1	0.001508866	100889927
Transcript_350913	lysozyme	4	0.001579517	587263
Transcript_291331	matrix metalloproteinase-24	2	0.001579939	577128
Transcript_228378	ADP-ribosylation factor-like protein 6	2	0.001582577	591507
Transcript_383671	anoctamin-8	1	0.001595851	588981
Transcript_456874	coiled-coil domain-containing protein 51	2	0.00167281	585678
Transcript_328102	protein Aster-B|GRAM domain-containing protein 1B	4	0.001674503	754516
Transcript_173387	HNF4G	1	0.001696184	574894
Transcript_453741	uncharacterized protein LOC100892807	3	0.001833911	100892807
Transcript_396224	sodium/glucose cotransporter 4	1	0.001869933	593500
Transcript_184289	F-box/LRR-repeat protein 7	1	0.001893942	580713
Transcript_435724	coiled-coil domain-containing protein 151	5	0.001915415	100893724
Transcript_278188	leucine-rich repeat-containing protein 15	1	0.001944018	100893380
Transcript_228502	S-crystallin SL11	1	0.001945235	586571
Transcript_430758	uncharacterized protein LOC100888279	1	0.001948734	100888279
Transcript_415734	peptidase M20 domain-containing protein 2	4	0.001958887	584225
Transcript_292151	uncharacterized protein LOC105437474	1	0.002019107	105437474
Transcript_359398	cytochrome P450 26A1|cytochrome P450 26B1	1	0.002045033	763716
Transcript_417667	ER membrane protein complex subunit 8|ER membrane protein complex subunit 8 pseudogene	3	0.002058001	582477
Transcript_363862	RNA-binding protein cabeza	1	0.00208612	105447104
Transcript_257591	fibrillin-2	1	0.002112952	581033
Transcript_224730	cytochrome c oxidase subunit 5B, mitochondrial	3	0.002126058	581274
Transcript_456276	heparan sulfate glucosamine 3-O-sulfotransferase 1	1	0.002149764	764585
Transcript_290553	isoamyl acetate-hydrolyzing esterase 1 homolog	4	0.002263181	591412
Transcript_324078	protein PBDC1|UPF0368 protein Cxorf26-like	2	0.00230034	577502
Transcript_236625	CDX1	1	0.002327014	584191
Transcript_309101	uncharacterized protein LOC105443655	3	0.002403175	105443655
Transcript_350196	ATP-binding cassette sub-family F member 2|ATP-binding cassette, sub-family F (GCN20), mem	2	0.002443557	581859
Transcript_204615	U6 snRNA-associated Sm-like protein LSm7	3	0.002487358	594039
Transcript_284394	EF-hand calcium-binding domain-containing protein 12	1	0.002516391	763537
Transcript_229121	LOW QUALITY PROTEIN: MMP37-like protein, mitochondrial|MMP37-like protein, mitochondria	2	0.002561667	575960
Transcript_310649	receptor-type guanylate cyclase gcy-8|insulin-like growth factor 1 receptor	1	0.002569395	591965

Transcript_239164	3-hydroxyanthranilate 3,4-dioxygenase	1	0.002593445	582154
Transcript_234886	multidrug resistance-associated protein 4|ATP-binding cassette, sub-family C (CFTR/MRP), mem	5	0.002593978	591982
Transcript_326960	uncharacterized protein LOC100891910	1	0.002627634	100891910
Transcript_186728	methylglutaconyl-CoA hydratase, mitochondrial	1	0.002651092	577505
Transcript_189831	L-gulonolactone oxidase	5	0.002653064	764246
Transcript_214947	calcium load-activated calcium channel|transmembrane and coiled-coil domain-containing prot	3	0.002669245	100892898
Transcript_444322	tubulin beta-4B chain	5	0.002730604	593362
Transcript_309473	autocrine proliferation repressor protein A	1	0.002840025	100889791
Transcript_254514	14-3-3 family protein artA|14-3-3 protein 3	2	0.002856679	581376
Transcript_187684	bcl-2 homologous antagonist/killer	2	0.002938261	588418
Transcript_54760	testis-specific serine/threonine-protein kinase 4-like	5	0.00297144	575157
Transcript_304049	uncharacterized aarF domain-containing protein kinase 1|putative aarF domain-containing prot	2	0.003065219	574821
Transcript_363577	uncharacterized protein LOC105438509	3	0.003084148	105438509
Transcript_340557	small nuclear ribonucleoprotein polypeptide E-like	3	0.003114499	580958
Transcript_182361	rho GTPase-activating protein 25	2	0.00313536	582939
Transcript_204566	ruvB-like 1	2	0.003144896	577255
Transcript_354492	presenilins-associated rhomboid-like protein, mitochondrial	2	0.003374583	576709
Transcript_374584	28S ribosomal protein S34, mitochondrial	2	0.003395751	753857
Transcript_208329	univin	3	0.003400545	373488
Transcript_243976	S-methylmethionine--homocysteine S-methyltransferase BHMT2-like	1	0.00340357	582542
Transcript_343639	glycine-rich cell wall structural protein 1|glycine-rich protein DOT1	2	0.003455777	764696
Transcript_236992	cyclic nucleotide-gated cation channel alpha-3	3	0.003662216	576815
Transcript_326935	deleted in malignant brain tumors 1 protein-like|neurotrypsin-like	1	0.003695293	762897
Transcript_445886	neurotrypsin	1	0.003720023	578211
Transcript_270797	uncharacterized protein LOC575472	1	0.003755055	575472
Transcript_456555	scavenger receptor cysteine-rich protein type 12	1	0.003772405	373431
Transcript_448686	fibrinogen C domain-containing protein 1-like	1	0.003887687	105440826
Transcript_450213	thioredoxin domain-containing protein 5	2	0.004022943	764720
Transcript_243778	UDP-glucuronosyltransferase 2C1	1	0.004132616	592958
Transcript_179967	condensin complex subunit 3-like	1	0.004145511	105447056
Transcript_187613	solute carrier family 28 member 3	1	0.004382769	574602
Transcript_280373	adenosylhomocysteinase	1	0.004452691	574714
Transcript_317804	uncharacterized protein LOC100891690	3	0.004527764	100891690
Transcript_438092	ATP-binding cassette, sub-family G (WHITE), member 2	1	0.004755746	578540
Transcript_356568	cystathionine gamma-lyase|putative cystathionine gamma-lyase 2	2	0.0051019	762952
Transcript_182233	nose resistant to fluoxetine protein 6	1	0.005139831	587932

Transcript_438569	acid-sensing ion channel 1A	1	0.005184244	575477
Transcript_314048	transmembrane 6 superfamily member 1	4	0.005222331	588706
Transcript_376909	5'-nucleotidase	1	0.005354264	753743
Transcript_368980	fumarylacetoacetate hydrolase domain-containing protein 2	5	0.005436549	763463
Transcript_271521	fos-related antigen 1	1	0.0054842	579838
Transcript_28806	queuine tRNA-ribosyltransferase catalytic subunit 1-like	2	0.00553486	579235
Transcript_319918	uncharacterized protein LOC100888641	1	0.005688136	100888641
Transcript_395198	synaptotagmin-14|synaptotagmin-16	5	0.005940282	577619
Transcript_330815	fibronectin-like	3	0.005999686	105447587
Transcript_271208	glycine-rich cell wall structural protein-like	4	0.006002727	105447344
Transcript_200340	uncharacterized protein YER152C	4	0.006272248	753534
Transcript_269278	HES4	1	0.006281806	592057
Transcript_443316	balbiani ring protein 3|prestalk protein|whey acidic protein-like	1	0.006407029	755393
Transcript_74981	DNA-directed RNA polymerases I, II, and III subunit RPABC3	2	0.006545429	591688
Transcript_308440	dimethylaniline monooxygenase [N-oxide-forming] 5	1	0.006648833	580981
Transcript_190979	leucine-rich repeat-containing protein 69	1	0.006656512	579382
Transcript_326137	LOW QUALITY PROTEIN: tRNA (adenine(37)-N6)-methyltransferase|nef-associated protein 1	3	0.006798408	577363
Transcript_462766	sodium-dependent multivitamin transporter	2	0.006889503	578787
Transcript_234973	nitric oxide-associated protein 1	2	0.006984558	576629
Transcript_437119	amassin-3	3	0.007112091	580528
Transcript_201140	ficolin-1-like	1	0.007272042	587736
Transcript_299780	rRNA methyltransferase 2, mitochondrial	3	0.00755268	582250
Transcript_315513	sushi domain-containing protein 2	1	0.007619095	580458
Transcript_443412	E3 ubiquitin-protein ligase MARCH8	3	0.007636877	579071
Transcript_282963	acetoacetyl-CoA synthetase	3	0.007722755	590598
Transcript_426847	retinol dehydrogenase 8	2	0.007781435	574716
Transcript_341560	cytochrome b-c1 complex subunit 6, mitochondrial	3	0.007857858	590393
Transcript_231242	ATP-citrate synthase	1	0.008053833	594785
Transcript_392370	methyltransferase-like protein 22	1	0.008061862	100894107
Transcript_50022	60S ribosomal protein L30	2	0.00815734	577852
Transcript_219767	lactase-phlorizin hydrolase	1	0.008398588	581938
Transcript_268430	ZNF260	4	0.008456303	100892543
Transcript_198022	P-selectin	2	0.008474676	105437801
Transcript_433401	proteasome assembly chaperone 3	2	0.008654302	100892637
Transcript_230273	dynein heavy chain 8, axonemal|dynein, axonemal, heavy chain 8	1	0.008769573	763294
Transcript_402513	acidic phospholipase A2|basic phospholipase A2 nigroxin A	1	0.00888625	757121

Transcript_326493	sodium-independent sulfate anion transporter	1	0.008989487	574650
Transcript_388333	ribonuclease H2 subunit C	3	0.009211094	752797
Transcript_462732	nesprin-1	5	0.009332795	105437706
Transcript_193119	titin|muscle M-line assembly protein unc-89|putative titin-like	3	0.00939858	590007
Transcript_210888	diamine acetyltransferase 2	3	0.009551632	100893060
Transcript_440549	uncharacterized protein LOC577038	1	0.010145783	577038
Transcript_48454	40S ribosomal protein S16	2	0.010706301	577040
Transcript_459460	THAP6	3	0.010762146	577120
Transcript_336905	uncharacterized protein LOC100888517	1	0.010835345	100888517
Transcript_437026	uncharacterized protein K02A2.6-like	5	0.010979249	105444440
Transcript_449725	calcineurin B homologous protein 1	2	0.010979249	576455
Transcript_354357	APOBEC1 complementation factor	1	0.011175702	575684
Transcript_258396	uncharacterized protein LOC588928	1	0.011267375	588928
Transcript_453256	WD repeat-containing protein 86	3	0.01200482	100888294
Transcript_226806	cytochrome P450 3A24	4	0.012022093	576854
Transcript_413396	3-ketoacyl-CoA thiolase A, peroxisomal	1	0.012424432	587557
Transcript_280723	scavenger receptor cysteine-rich protein	1	0.012532041	373211
Transcript_61404	dynein heavy chain 5, axonemal	1	0.012843572	577371
Transcript_13256	protein disulfide-isomerase A5	5	0.013269666	576717
Transcript_292279	dnaJ homolog subfamily C member 28	1	0.013626876	587875
Transcript_256585	microfibril-associated glycoprotein 4	1	0.013830912	582445
Transcript_453252	N-acetyltransferase 9-like protein	2	0.014015317	586164
Transcript_374389	alpha-(1,3)-fucosyltransferase 6	4	0.014272136	579774
Transcript_265355	protein boule-like|bromodomain-containing protein 4-like	1	0.014681263	100892519
Transcript_297498	EH domain-containing protein 1|EH domain-containing protein 3-like	2	0.014744428	587455
Transcript_204631	tyrosine-protein kinase receptor Tie-2-like	1	0.014745656	105439696
Transcript_352756	protein TFG	3	0.014753533	594769
Transcript_400118	uncharacterized protein LOC100893690	1	0.015012477	100893690
Transcript_451319	40S ribosomal protein S12	2	0.015117118	590740
Transcript_440408	N-fatty-acyl-amino acid synthase/hydrolase PM20D1|probable carboxypeptidase PM20D1|puta	4	0.015510861	579513
Transcript_285315	N-alpha-acetyltransferase 40	2	0.015578148	577667
Transcript_361820	L-threonine 3-dehydrogenase, mitochondrial|inactive L-threonine 3-dehydrogenase, mitochond	2	0.015611387	584440
Transcript_189682	peroxisomal carnitine O-octanoyltransferase	1	0.015664898	585404
Transcript_214210	late histone H2A.L3	3	0.015709704	373348
Transcript_367907	uncharacterized protein LOC586472|Small GTPase superfamily domain containing protein	1	0.015758585	586472
Transcript_393644	SAFB-like transcription modulator	2	0.01587077	585734

Transcript_257884	uncharacterized protein LOC583434|urease	1	0.016315729	583434
Transcript_195199	arylsulfatase	1	0.016536437	582391
Transcript_257979	echinoderm microtubule-associated protein-like 4|uncharacterized protein LOC581284	1	0.016740806	581284
Transcript_321307	mitochondrial fission regulator 2|mitochondrial fission regulator 1	3	0.016869408	764790
Transcript_240999	cyclin-dependent kinases regulatory subunit	1	0.017248255	100891492
Transcript_2061	mitochondrial ATP synthase alpha subunit precursor	2	0.018413908	373382
Transcript_355725	protein disulfide-isomerase A3	2	0.018926929	577673
Transcript_258178	zinc finger protein 474-like	5	0.01959868	100888783
Transcript_225983	histamine N-methyltransferase	4	0.019730461	582038
Transcript_388758	uncharacterized protein LOC591506|UPF0394 inner membrane protein yeeE-like	1	0.020558406	591506
Transcript_252605	cation-dependent mannose-6-phosphate receptor-like	1	0.020779714	105438545
Transcript_307974	malignant fibrous histiocytoma-amplified sequence 1 homolog	3	0.020863101	100888805
Transcript_325168	zonadhesin-like	1	0.021381996	105438765
Transcript_269460	probable D-lactate dehydrogenase, mitochondrial|lactate dehydrogenase D	4	0.021486102	591812
Transcript_256749	peptidyl-prolyl cis-trans isomerase G|serine/arginine repetitive matrix protein 2-like	1	0.021654179	100890356
Transcript_258900	KRP170	4	0.021974774	373241
Transcript_386762	uncharacterized protein LOC100892326	4	0.022071669	100892326
Transcript_392136	somatostatin receptor type 2	1	0.022255228	100889243
Transcript_405707	ADP-ribosyl cyclase|ADP-ribosyl cyclase beta	1	0.022643114	580868
Transcript_280991	phytanoyl-CoA dioxygenase domain-containing protein 1	1	0.022977014	753147
Transcript_314947	microfibril-associated glycoprotein 4-like	3	0.022996409	581168
Transcript_396061	putative ISG12 protein	1	0.023872748	403119
Transcript_412518	ATP-dependent (S)-NAD(P)H-hydrate dehydratase	1	0.025975248	586141
Transcript_246157	LOW QUALITY PROTEIN: tubulin beta chain	5	0.026130789	594231
Transcript_351435	medium-chain acyl-CoA ligase ACSF2, mitochondrial-like|acyl-CoA synthetase family member 2,	1	0.02642635	593582
Transcript_200945	cathepsin L1	3	0.027167837	575203
Transcript_308628	protein PRY1|golgi-associated plant pathogenesis-related protein 1	1	0.027167837	100893059
Transcript_398823	uncharacterized protein LOC589927|CUB domain-containing protein	1	0.027751362	589927
Transcript_343503	LOW QUALITY PROTEIN: very early blastula protein 4|very early blastula protein 4	5	0.02946305	373489
Transcript_194733	uncharacterized protein LOC579958	1	0.029757117	579958
Transcript_241860	denticleless protein homolog	4	0.030087247	584715
Transcript_265286	tumor suppressor candidate 2	2	0.030240058	594794
Transcript_254071	actin, cytoskeletal 3B|actin, cytoskeletal 3	3	0.03034686	100890099
Transcript_459535	transmembrane protein 187	4	0.031438231	100887935
Transcript_193614	somatostatin receptor type 5	2	0.031787754	105444813
Transcript_386066	zinc finger protein 236	2	0.031981685	100890022

Transcript_407578	solute carrier family 23 member 1	1	0.032288234	581718
Transcript_373383	uncharacterized protein LOC581986|proteinase T	1	0.032789184	581986
Transcript_284740	uncharacterized protein C16orf96 homolog|uncharacterized protein C16orf96|uncharacterized	5	0.033005953	755388
Transcript_57336	profilin|spCoel1	3	0.034328683	373409
Transcript_299519	uncharacterized protein LOC105447398	2	0.034769137	105447398
Transcript_289174	annexin A7	3	0.035301747	764615
Transcript_453447	NAD kinase	3	0.035404269	584340
Transcript_346874	alpha-tocopherol transfer protein-like	3	0.035599419	575290
Transcript_324882	uncharacterized protein LOC105440703	3	0.035609898	105440703
Transcript_375307	uncharacterized protein LOC100888794	1	0.037345332	100888794
Transcript_285525	uncharacterized protein LOC100891603	3	0.037526216	100891603
Transcript_315280	meiosis 1 arrest protein	1	0.037581022	100893539
Transcript_54635	uncharacterized protein LOC100891675	3	0.03765683	100891675
Transcript_359165	calponin homology domain-containing protein DDB_G0272472|intracellular protein transport p	1	0.037852399	100890558
Transcript_281352	centrosomal protein of 290 kDa-like	2	0.038176421	105442844
Transcript_117530	D(2) dopamine receptor A-like	1	0.038798225	105439410
Transcript_82652	mitochondrial ribosome-associated GTPase 1	4	0.039178574	588789
Transcript_427415	paraneoplastic antigen Ma3-like	2	0.039625395	105447116
Transcript_345735	DLX5	3	0.04058574	593496
Transcript_218552	cytosolic beta-glucosidase	1	0.04098007	589416
Transcript_322346	triple QxxK/R motif-containing protein-like|acyl-CoA synthetase family member 3, mitochondria	4	0.041155658	105440874
Transcript_300712	protein DDI1 homolog 2	2	0.04169834	590177
Transcript_67216	cGMP-dependent protein kinase 1	2	0.042140028	589462
Transcript_390895	vesicular inhibitory amino acid transporter-like	1	0.043654839	755450
Transcript_366842	bindin	5	0.044607927	373276
Transcript_207308	uncharacterized protein LOC587864	1	0.045911275	587864
Transcript_177363	LOW QUALITY PROTEIN: aromatic-L-amino-acid decarboxylase	1	0.04611209	577767
Transcript_371808	dexamethasone-induced Ras-related protein 1	3	0.04725451	578089
Transcript_206242	uncharacterized protein LOC756858|Solute-binding protein family 3/N-terminal domain of Mltl	1	0.047665727	756858
Transcript_64977	40S ribosomal protein S15	3	0.048248049	574998
Transcript_364074	LOW QUALITY PROTEIN: zinc finger protein 708-like	2	0.048728883	593726

Table S19. Functional analysis of coexpressed clusters

Cluster	Category	Term	Count	%	PValue	Genes	List Total	Pop Hits	Pop Total	Fold Enrichment	Bonferroni	Benjamini	FDR
1	Biological Process	GO:0044699~single-organism process	23	7.565789474	2.70E-04	591943, 591159, 592958, 755450, 100891492, 577570, 757121, 581938, 105439410, 577952, 580868,	28	81	162	1.642857143	0.086471814	0.090428842	0.090428842
	Biological Process	GO:0044763~single-organism cellular process	20	6.578947368	0.006625553	591943, 591159, 592958, 755450, 100891492, 577570, 757121, 105439410, 577952, 580868, 1008892	28	76	162	1.522556391	0.892142638	1	1
	Biological Process	GO:0005975~carbohydrate metabolic process	4	1.315789474	0.033087446	586329, 586832, 581938, 583563	28	5	162	4.628571429	0.999987273	1	1
	Biological Process	GO:0044710~single-organism metabolic process	9	2.960526316	0.050405274	100891893, 592958, 586329, 574714, 577570, 757121, 581938, 583563, 100891910	28	27	162	1.928571429	0.99999997	1	1
	Biological Process	GO:0044707~single-multicellular organism process	4	1.315789474	0.058741299	591159, 373360, 580173, 105439410	28	6	162	3.857142857	0.999999998	1	1
	Biological Process	GO:0098609~cell-cell adhesion	3	0.986842105	0.072334369	591943, 591159, 105440441	28	3	162	5.785714286	1	1	1
	Biological Process	GO:0006082~organic acid metabolic process	5	1.644736842	0.088248913	100891893, 592958, 574714, 577570, 100891910	28	11	162	2.62987013	1	1	1
	Biological Process	GO:0032501~multicellular organismal process	4	1.315789474	0.091301958	591159, 373360, 580173, 105439410	28	7	162	3.306122449	1	1	1
	Biological Process	GO:0022610~biological adhesion	4	1.315789474	0.091301958	591943, 591159, 576823, 105440441	28	7	162	3.306122449	1	1	1
	Biological Process	GO:0007155~cell adhesion	4	1.315789474	0.091301958	591943, 591159, 576823, 105440441	28	7	162	3.306122449	1	1	1
	Cellular Component	GO:0016020~membrane	28	9.210526316	3.25E-08	591159, 373211, 373431, 755450, 585092, 578540, 100893380, 105439410, 592585, 105437474, 5769	38	61	183	2.210526316	2.18E-06	1.25E-06	1.21E-06
	Cellular Component	GO:0031224~intrinsic component of membrane	25	8.223684211	3.73E-08	591159, 755450, 578540, 100893380, 105439410, 592585, 105437474, 576910, 582219, 586832, 1054	38	49	183	2.457035446	2.50E-06	1.25E-06	1.21E-06
	Cellular Component	GO:0016021~integral component of membrane	24	7.894736842	1.79E-07	591943, 586672, 591159, 592958, 755450, 578540, 100893380, 100888815, 105439410, 105437474, 5	38	48	183	2.407894737	1.20E-05	3.99E-06	3.87E-06
	Cellular Component	GO:0044425~membrane part	25	8.223684211	5.07E-07	591159, 755450, 578540, 100893380, 105439410, 592585, 105437474, 576910, 582219, 586832, 1054	38	54	183	2.229532164	3.40E-05	8.49E-06	8.24E-06
	Cellular Component	GO:0071944~cell periphery	6	1.973684211	0.017738484	580868, 100889243, 591159, 582219, 105440441, 589852	38	9	183	3.210526316	0.698548349	0.198079733	0.192166905
	Cellular Component	GO:0005886~plasma membrane	6	1.973684211	0.017738484	580868, 100889243, 591159, 582219, 105440441, 589852	38	9	183	3.210526316	0.698548349	0.198079733	0.192166905
	Molecular Function	GO:0060089~molecular transducer activity	8	2.631578947	0.002391091	100889243, 591159, 576823, 373431, 373211, 585092, 105439410, 100888815	46	10	183	3.182608696	0.318209368	0.191287272	0.191287272
	Molecular Function	GO:0004872~receptor activity	8	2.631578947	0.002391091	100889243, 591159, 576823, 373431, 373211, 585092, 105439410, 100888815	46	10	183	3.182608696	0.318209368	0.191287272	0.191287272
	Molecular Function	GO:0038024~cargo receptor activity	4	1.315789474	0.046606505	576823, 373431, 373211, 585092	46	4	183	3.97826087	0.999517442	1	1
	Molecular Function	GO:0005044~scavenger receptor activity	4	1.315789474	0.046606505	576823, 373431, 373211, 585092	46	4	183	3.97826087	0.999517442	1	1
	Molecular Function	GO:0043167~ion binding	12	3.947368421	0.060239568	591943, 575697, 100891893, 373360, 577570, 757121, 585092, 580652, 105440441, 592971, 592585,	46	29	183	1.646176912	0.999951834	1	1
	Molecular Function	GO:0005509~calcium ion binding	5	1.644736842	0.063060918	591943, 373360, 757121, 105440441, 592971	46	7	183	2.841614907	0.999970227	1	1
	Molecular Function	GO:0003824~catalytic activity	27	8.881578947	0.067218842	591159, 577570, 585092, 578540, 577198, 592585, 574714, 585404, 373360, 580652, 586832, 588773	46	86	183	1.248988878	0.999985386	1	1
	Molecular Function	GO:0016787~hydrolase activity	14	4.605263158	0.07561082	591159, 577570, 757121, 578540, 581938, 592585, 580868, 576823, 574714, 373360, 583337, 580652	46	37	183	1.505287897	0.999996558	1	1
	Molecular Function	GO:0043169~cation binding	11	3.618421053	0.086149331	591943, 575697, 100891893, 373360, 577570, 757121, 585092, 580652, 105440441, 592971, 592585	46	27	183	1.620772947	0.99999945	1	1
	Molecular Function	GO:0046872~metal ion binding	11	3.618421053	0.086149331	591943, 575697, 100891893, 373360, 577570, 757121, 585092, 580652, 105440441, 592971, 592585	46	27	183	1.620772947	0.99999945	1	1
	Molecular Function	GO:0016788~hydrolase activity, acting on ester bonds	4	1.315789474	0.096329578	591159, 757121, 580652, 580300	46	5	183	3.182608696	0.999999908	1	1
	Molecular Function	GO:0008237~metallopeptidase activity	4	1.315789474	0.096329578	373360, 577570, 588773, 592585	46	5	183	3.182608696	0.999999908	1	1
2	Biological Process	GO:1901566~organonitrogen compound biosynthetic proce	23	7.324840764	9.44E-04	593029, 593744, 575080, 591935, 588945, 577040, 762952, 591341, 593861, 577020, 591498, 590740	58	38	162	1.690562613	0.378755347	0.475805574	0.475805574
	Biological Process	GO:0044271~cellular nitrogen compound biosynthetic proc	26	8.280254777	0.003317447	752563, 588945, 591935, 577255, 591498, 590740, 574895, 575225, 584839, 581865, 100892475, 373	58	48	162	1.512931034	0.812649242	0.835996592	0.835996592
	Biological Process	GO:1901564~organonitrogen compound metabolic process	23	7.324840764	0.00631878	593029, 593744, 575080, 591935, 588945, 577040, 762952, 591341, 593861, 577020, 591498, 590740	58	42	162	1.52955665	0.959024032	1	1
	Biological Process	GO:0044249~cellular biosynthetic process	28	8.917197452	0.013615712	752563, 754268, 588945, 591935, 577255, 591498, 590740, 574895, 575225, 584839, 581865, 100892	58	57	162	1.372050817	0.999001708	1	1
	Biological Process	GO:0043043~peptide biosynthetic process	16	5.095541401	0.014924636	593029, 593744, 575080, 577040, 591341, 593861, 591498, 590740, 373469, 577852, 574895, 575225	58	27	162	1.655172414	0.999488781	1	1
	Biological Process	GO:0006412~translation	16	5.095541401	0.014924636	593029, 593744, 575080, 577040, 591341, 593861, 591498, 590740, 373469, 577852, 574895, 575225	58	27	162	1.655172414	0.999488781	1	1
	Biological Process	GO:0043604~amide biosynthetic process	16	5.095541401	0.014924636	593029, 593744, 575080, 577040, 591341, 593861, 591498, 590740, 373469, 577852, 574895, 575225	58	27	162	1.655172414	0.999488781	1	1
	Biological Process	GO:0009058~biosynthetic process	28	8.917197452	0.01872805	752563, 754268, 588945, 591935, 577255, 591498, 590740, 574895, 575225, 584839, 581865, 100892	58	58	162	1.348394768	0.999927247	1	1
	Biological Process	GO:1901576~organic substance biosynthetic process	28	8.917197452	0.01872805	752563, 754268, 588945, 591935, 577255, 591498, 590740, 574895, 575225, 584839, 581865, 100892	58	58	162	1.348394768	0.999927247	1	1
	Biological Process	GO:0006518~peptide metabolic process	16	5.095541401	0.034271009	593029, 593744, 575080, 577040, 591341, 593861, 591498, 590740, 373469, 577852, 574895, 575225	58	29	162	1.541022592	0.999999977	1	1
	Biological Process	GO:0043603~cellular amide metabolic process	16	5.095541401	0.049078681	593029, 593744, 575080, 577040, 591341, 593861, 591498, 590740, 373469, 577852, 574895, 575225	58	30	162	1.489655172	1	1	1
	Biological Process	GO:0034645~cellular macromolecule biosynthetic process	21	6.687898089	0.0528505	591688, 752563, 593029, 754268, 593744, 575080, 577040, 591341, 593861, 577255, 591498, 590740	58	43	162	1.364073777	1	1	1
	Biological Process	GO:0009059~macromolecule biosynthetic process	21	6.687898089	0.0528505	591688, 752563, 593029, 754268, 593744, 575080, 577040, 591341, 593861, 577255, 591498, 590740	58	43	162	1.364073777	1	1	1
	Biological Process	GO:0034641~cellular nitrogen compound metabolic proces	32	10.1910828	0.070295795	592405, 752563, 754268, 579591, 588945, 591935, 577255, 591498, 590740, 582919, 574895, 575225	58	74	162	1.207828518	1	1	1
	Biological Process	GO:0006807~nitrogen compound metabolic process	33	10.50955414	0.073219103	592405, 752563, 754268, 579591, 588945, 591935, 577255, 591498, 590740, 582919, 574895, 575225	58	77	162	1.197044335	1	1	1
	Cellular Component	GO:0005840~ribosome	14	4.458598726	2.43E-04	593029, 593744, 577040, 580091, 591341, 593861, 591498, 590740, 373469, 577852, 574895, 584839	59	18	183	2.412429379	0.033490967	0.03406049	0.032357465
	Cellular Component	GO:0044391~ribosomal subunit	10	3.184713376	0.001873756	577852, 593029, 581865, 593744, 577040, 591341, 593861, 587414, 591498, 590740	59	12	183	2.584745763	0.230928925	0.131162907	0.124604762
	Cellular Component	GO:0005829~cytosol	10	3.184713376	0.004539866	577852, 581865, 593744, 588945, 577040, 580091, 591341, 577020, 593861, 590740	59	13	183	2.385919166	0.471138308	0.139107249	0.132151887
	Cellular Component	GO:0022626~cytosolic ribosome	8	2.547770701	0.004968116	577852, 581865, 593744, 577040, 580091, 591341, 593861, 590740	59	9	183	2.757062147	0.502057038	0.139107249	0.132151887
	Cellular Component	GO:0044445~cytosolic part	8	2.547770701	0.004968116	577852, 581865, 593744, 577040, 580091, 591341, 593861, 590740	59	9	183	2.757062147	0.502057038	0.139107249	0.132151887
	Cellular Component	GO:0030529~intracellular ribonucleoprotein complex	18	5.732484076	0.010999297	593029, 587044, 593744, 575080, 577040, 580091, 591341, 593861, 591498, 590740, 373469, 582919	59	34	183	1.642073779	0.787419209	0.185105101	0.175849846
	Cellular Component	GO:1990904~ribonucleoprotein complex	18	5.732484076	0.010999297	593029, 587044, 593744, 575080, 577040, 580091, 591341, 593861, 591498, 590740, 373469, 582919	59	34	183	1.642073779	0.787419209	0.185105101	0.175849846
	Cellular Component	GO:0043228~non-membrane-bounded organelle	23	7.324840764	0.012551192	591688, 593029, 587044, 581030, 593744, 577040, 580091, 373275, 591341, 593861, 577255, 591498	59	48	183	1.486228814	0.829375031	0.185105101	0.175849846
	Cellular Component	GO:0043232~intracellular non-membrane-bounded organel	23	7.324840764	0.012551192	591688, 593029, 587044, 581030, 593744, 577040, 580091, 373275, 591341, 593861, 577255, 591498	59	48	183	1.486228814	0.829375031	0.185105101	0.175849846
	Cellular Component	GO:0015934~large ribosomal subunit	7	2.229299363	0.013221793	577852, 593029, 581865, 591341, 593861, 587414, 591498	59	8	183	2.713983051	0.844855302	0.185105101	0.175849846
	Cellular Component	GO:0044444~cytoplasmic part	24	7.643312102	0.018000281	593029, 593744, 575080, 588945, 588418, 577040, 580091, 591341, 593861, 577020, 373418, 591498	59	52	183	1.431551499	0.921369521	0.229094479	0.217639756
	Cellular Component	GO:0005737~cytoplasm	30	9.554140127	0.025748533	588945, 373275, 373418, 591498, 590740, 574660, 574895, 575225, 584839, 581865, 575808, 593029	59	71	183	1.310575316	0.974061219	0.300399556	0.285379578
	Cellular Component	GO:0005622~intracellular	47	14.96815287	0.064294176	592405, 752563, 754268, 581030, 579591, 588945, 373275, 373418, 763814, 577255, 591498, 590740	59	130	183	1.121382008	0.999908902	0.64317898	0.611020031
	Cellular Component	GO:0005623~cell	51	16.24203822	0.064317898	373418, 591498, 591178, 590165, 584839, 100892475, 373382, 752213, 575808, 591688, 593029, 587	59	144	183	1.098516949	0.999909225	0.64317898	0.611020031
	Cellular Component	GO:0044424~intracellular part	45	14.33121019	0.075363329	592405, 752563, 754268, 581030, 579591, 588945, 373275, 373418, 577255, 591498, 590740, 574660	59	124	183	1.12561509	0.999982783	0.703391071	0.668221517
	Cellular Component	GO:0032991~macromolecular complex	26	8.280254777	0.080766921	581030, 577255, 591498, 590740, 574660, 582919, 574895, 584839, 581865, 100892475, 373382, 752	59	64	183	1.260063559	0.999992421	0.706710556	0.671375028
	Cellular Component	GO:0022625~cytosolic large ribosomal subunit	4	1.27388535	0.094691349	577852, 581865, 591341, 593861	59	4	183	3.101694915	0.999999106	0.779811106	0.740820551
	Molecular Function	GO:0005198~structural molecule activity	17	5.414012739	3.17E-05	593029, 593744, 577040, 373275, 591341, 593861, 591498, 590740, 373469, 577852, 574895, 575225	63	21	183	2.351473923	0.005981249	0.005999114	0.005999114
	Molecular Function	GO:0003735~structural constituent of ribosome	15	4.777070064	7.91E-05	593029, 593744, 577040, 591341, 593861, 591498, 590740, 373469, 577852, 574895, 575225, 584839	63	18	183	2.420634921	0.014841786	0.007476218	0.007476218
	Biological Process	GO:0071840~cellular component organization or biogenesis	26	10.6122449	0.008717447	587575, 589835, 585590, 592267, 575126, 574998, 105438529, 577928, 589486, 582011, 580493, 588	61	48	162	1.43852459	0.994677905	1	1
	Biological Process	GO:0044260~cellular macromolecule metabolic process	37	15.10204082	0.01136628	587575, 590942, 586283, 590968, 575790, 592267, 593496, 578762, 578317, 575203, 580958, 575347	61	77	162	1.276133702	0.998925564	1	1
	Biological Process	GO:0043170~macromolecule metabolic process	37	15.10204082	0.01136628	587575, 590942, 586283, 590968, 575790, 592267, 593496, 578762, 578317, 575203, 580958, 575347	61	77	162	1.276133702	0.998925564	1	1
	Biological Process	GO:0016070~RNA metabolic process	18	7.346938776	0.020049679	588854, 752151, 577044, 594039, 575790, 373348, 592267, 593496, 578317, 577954, 575632, 580958	61	31	162	1.542041248	0.999994504	1	1
	Biological Process	GO:0006508~proteolysis	8	3.265306122	0.031373568	590942, 591601, 575334, 575203, 575347, 100889149, 585921, 578762	61	10	162	2.124590164	0.999999995	1	1
	Biological Process	GO:0051641~cellular localization	8	3.265306122	0.031373568	575632, 589835, 585590, 585934, 589195, 577928, 591499, 581274	61	10	162	2.124590164	0.999999995	1	1
	Biological Process	GO:0009057~macromolecule catabolic process	8	3.265306122	0.031373568	590942, 591601, 575334, 575203, 752151, 100889149, 585921, 578762	61	10	162	2.124590164	0.999999995	1	1
	Biological Process	GO:0090304~nucleic acid metabolic process	20	8.163265306	0.032613223	588854, 590968, 752151, 577044, 594039, 575790, 373348, 592267, 593496, 578317, 577954, 575632	61	37	162	1.435533895	0.999999998	1	1

	Biological Process	GO:0006139~nucleobase-containing compound metabolic p	24	9.795918367	0.034737512	584340, 588854, 590968, 752151, 577044, 594039, 575790, 373348, 592267, 593496, 578317, 574069	61	47	162	1.356121381	0.999999999	1	1
	Biological Process	GO:0046483~heterocycle metabolic process	24	9.795918367	0.047021722	584340, 588854, 590968, 752151, 577044, 594039, 575790, 373348, 592267, 593496, 578317, 574069	61	48	162	1.327868852	1	1	1
	Biological Process	GO:0022613~ribonucleoprotein complex biogenesis	14	5.714285714	0.050722687	587575, 588854, 585590, 577044, 592267, 577954, 575632, 575126, 574998, 105438529, 589486, 582	61	24	162	1.549180328	1	1	1
	Biological Process	GO:0016043~cellular component organization	18	7.346938776	0.060468956	587575, 588415, 589835, 585590, 373348, 373503, 580307, 763690, 580528, 583958, 585934, 589392	61	34	162	1.405978785	1	1	1
	Biological Process	GO:0044248~cellular catabolic process	8	3.265306122	0.060621815	590942, 591601, 575334, 575203, 752151, 100889149, 585921, 578762	61	11	162	1.931445604	1	1	1
	Biological Process	GO:0010467~gene expression	26	10.6122449	0.060796025	587575, 586283, 575790, 593496, 578317, 580958, 574998, 576519, 105438529, 589486, 582011, 580	61	54	162	1.278688525	1	1	1
	Biological Process	GO:0006725~cellular aromatic compound metabolic proces	24	9.795918367	0.062339684	584340, 588854, 590968, 752151, 577044, 594039, 575790, 373348, 592267, 593496, 578317, 574069	61	49	162	1.300769488	1	1	1
	Biological Process	GO:1990542~mitochondrial transmembrane transport	5	2.040816327	0.06318405	589835, 585934, 589195, 577928, 581274	61	5	162	2.655737705	1	1	1
	Biological Process	GO:0006839~mitochondrial transport	5	2.040816327	0.06318405	589835, 585934, 589195, 577928, 581274	61	5	162	2.655737705	1	1	1
	Biological Process	GO:0030163~protein catabolic process	7	2.857142857	0.064304928	590942, 591601, 575334, 575203, 100889149, 585921, 578762	61	9	162	2.06557377	1	1	1
	Biological Process	GO:0051649~establishment of localization in cell	7	2.857142857	0.064304928	575632, 589835, 585590, 585934, 589195, 577928, 581274	61	9	162	2.06557377	1	1	1
	Biological Process	GO:0046907~intracellular transport	7	2.857142857	0.064304928	575632, 589835, 585590, 585934, 589195, 577928, 581274	61	9	162	2.06557377	1	1	1
	Biological Process	GO:0044265~cellular macromolecule catabolic process	7	2.857142857	0.064304928	590942, 591601, 575334, 575203, 752151, 585921, 578762	61	9	162	2.06557377	1	1	1
	Biological Process	GO:0042254~ribosome biogenesis	11	4.489795918	0.072996416	575632, 577954, 588854, 575126, 585590, 574998, 577044, 589486, 580493, 592267, 586103	61	18	162	1.62295082	1	1	1
	Biological Process	GO:0006996~organelle organization	14	5.714285714	0.073674626	588415, 589835, 585590, 373348, 373503, 763690, 580528, 583958, 585934, 589392, 574998, 589195	61	25	162	1.487213115	1	1	1
	Biological Process	GO:0044085~cellular component biogenesis	16	6.530612245	0.079112304	587575, 588854, 585590, 577044, 592267, 373503, 577954, 575632, 580528, 575126, 574998, 105438	61	30	162	1.416393443	1	1	1
	Biological Process	GO:1901360~organic cyclic compound metabolic process	24	9.795918367	0.081036534	584340, 588854, 590968, 752151, 577044, 594039, 575790, 373348, 592267, 593496, 578317, 574069	61	50	162	1.274754098	1	1	1
	Biological Process	GO:0006396~RNA processing	10	4.081632653	0.081966607	575632, 577954, 588854, 580958, 105438529, 577044, 594039, 589486, 580493, 586103	61	16	162	1.659836066	1	1	1
	Biological Process	GO:0034641~cellular nitrogen compound metabolic proces	33	13.46938776	0.090703903	584340, 587575, 586283, 590968, 575790, 592267, 593496, 578317, 580958, 575347, 105443655, 574	61	74	162	1.184315463	1	1	1
3	Biological Process	GO:0044238~primary metabolic process	41	16.73469388	0.095328282	584340, 587575, 590942, 586283, 590968, 575790, 592267, 593496, 578762, 578317, 575203, 580958	61	96	162	1.134221311	1	1	1
	Cellular Component	GO:0044428~nuclear part	18	7.346938776	3.36E-05	591601, 752151, 577044, 594039, 373348, 592267, 591574, 578317, 577954, 575632, 575126, 580958	67	22	183	2.234735414	0.0060654	0.006083767	0.005848483
	Cellular Component	GO:0031981~nuclear lumen	14	5.714285714	0.001168745	752151, 577044, 373348, 592267, 591574, 578317, 577954, 575632, 575126, 576519, 583455, 589486	67	18	183	2.124378109	0.190765557	0.091662373	0.08811742
	Cellular Component	GO:0043234~protein complex	24	9.795918367	0.001519266	587575, 590942, 591601, 589835, 586283, 752151, 574071, 594039, 373348, 578762, 578317, 575334	67	40	183	1.63880597	0.240576906	0.091662373	0.08811742
	Cellular Component	GO:0032991~macromolecular complex	33	13.46938776	0.003446902	587575, 590942, 589835, 586283, 592267, 578762, 578317, 580958, 574998, 585921, 576519, 105438	67	64	183	1.408348881	0.464720854	0.124304455	0.1194971
	Cellular Component	GO:0031974~membrane-enclosed lumen	17	6.93877551	0.00401343	588415, 589835, 752151, 577044, 373348, 592267, 591574, 578317, 577954, 575632, 575126, 575347	67	26	183	1.785878301	0.51707435	0.124304455	0.1194971
	Cellular Component	GO:0005622~intracellular	56	22.85714286	0.00412059	590968, 575290, 579451, 591499, 592785, 592267, 591574, 578762, 578317, 575126, 575203, 580958	67	130	183	1.176578645	0.526388345	0.124304455	0.1194971
	Cellular Component	GO:0005730~nucleolus	11	4.489795918	0.005775559	578317, 575632, 577954, 575126, 583455, 577044, 589486, 580493, 592267, 591574, 586103	67	14	183	2.146055437	0.649504234	0.149339452	0.143563893
	Cellular Component	GO:0070013~intracellular organelle lumen	15	6.12244898	0.008795471	588415, 752151, 577044, 373348, 592267, 591574, 578317, 577954, 575632, 575126, 576519, 583455	67	23	183	1.781310837	0.797906061	0.17688669	0.170045769
	Cellular Component	GO:0043233~organelle lumen	15	6.12244898	0.008795471	588415, 752151, 577044, 373348, 592267, 591574, 578317, 577954, 575632, 575126, 576519, 583455	67	23	183	1.781310837	0.797906061	0.17688669	0.170045769
	Cellular Component	GO:0005634~nucleus	24	9.795918367	0.013239748	591601, 588854, 590968, 752151, 578771, 577044, 594039, 579451, 373348, 592267, 593496, 591574	67	45	183	1.456716418	0.910399978	0.203917025	0.196030731
	Cellular Component	GO:0043231~intracellular membrane-bounded organelle	36	14.69387755	0.013519361	589835, 590968, 579451, 592785, 592267, 593496, 591574, 578317, 575126, 575203, 580958, 575347	67	76	183	1.293794187	0.914880224	0.203917025	0.196030731
	Cellular Component	GO:0043227~membrane-bounded organelle	36	14.69387755	0.013519361	589835, 590968, 579451, 592785, 592267, 593496, 591574, 578317, 575126, 575203, 580958, 575347	67	76	183	1.293794187	0.914880224	0.203917025	0.196030731
	Cellular Component	GO:0005615~extracellular space	7	2.857142857	0.02670786	580528, 575203, 373488, 100889149, 373196, 585147, 373503	67	8	183	2.389925373	0.9925523	0.328818067	0.316101346
	Cellular Component	GO:0044421~extracellular region part	7	2.857142857	0.02670786	580528, 575203, 373488, 100889149, 373196, 585147, 373503	67	8	183	2.389925373	0.9925523	0.328818067	0.316101346
	Cellular Component	GO:0044424~intracellular part	52	21.2244898	0.027250116	590968, 579451, 591499, 592785, 592267, 591574, 578762, 578317, 575126, 575203, 580958, 574998	67	124	183	1.145402022	0.993266902	0.328818067	0.316101346
	Cellular Component	GO:0005623~cell	58	23.67346939	0.040687985	590968, 575290, 579451, 591499, 592785, 592267, 591574, 578762, 578317, 575126, 575203, 580958	67	144	183	1.100124378	0.999457076	0.460282831	0.442481838
	Cellular Component	GO:0044422~organelle part	29	11.83673469	0.047921827	589835, 592785, 592267, 591574, 578317, 575126, 580958, 575347, 574998, 576519, 105438529, 577	67	62	183	1.277563794	0.999862043	0.481880589	0.463244323
	Cellular Component	GO:0044446~intracellular organelle part	29	11.83673469	0.047921827	589835, 592785, 592267, 591574, 578317, 575126, 580958, 575347, 574998, 576519, 105438529, 577	67	62	183	1.277563794	0.999862043	0.481880589	0.463244323
	Cellular Component	GO:0005576~extracellular region	7	2.857142857	0.057043871	580528, 575203, 373488, 100889149, 373196, 585147, 373503	67	9	183	2.124378109	0.999975848	0.543417932	0.522401769
	Cellular Component	GO:0044464~cell part	56	22.85714286	0.071033853	590968, 575290, 579451, 591499, 592785, 592267, 591574, 578762, 578317, 575126, 575203, 580958	67	140	183	1.092537313	0.999998386	0.642856373	0.617994525
	Molecular Function	GO:0003676~nucleic acid binding	21	8.571428571	3.97E-04	587575, 586283, 752151, 574071, 589939, 578771, 577044, 594039, 577120, 373348, 593496, 578317	55	37	183	1.888452088	0.064153823	0.066290996	0.066290996
	Molecular Function	GO:0003723~RNA binding	14	5.714285714	0.002018721	587575, 586283, 752151, 574071, 578771, 577044, 594039, 580307, 583958, 100893477, 574998, 105	55	22	183	2.117355372	0.286424619	0.168563229	0.168563229
	Molecular Function	GO:1901363~heterocyclic compound binding	30	12.24489796	0.004679523	587575, 586283, 590968, 589939, 575790, 593496, 577721, 578317, 575126, 575347, 105443655, 574	55	70	183	1.425974026	0.543110853	0.260493427	0.260493427
	Molecular Function	GO:0097159~organic cyclic compound binding	30	12.24489796	0.006280826	587575, 586283, 590968, 589939, 575790, 593496, 577721, 578317, 575126, 575347, 105443655, 574	55	71	183	1.405889885	0.650833305	0.262224501	0.262224501
	Molecular Function	GO:0008135~translation factor activity, RNA binding	7	2.857142857	0.066618535	587575, 580307, 583958, 586283, 574071, 578771, 582011	55	11	183	2.117355372	0.999990003	1	1
	Molecular Function	GO:0008565~protein transporter activity	4	1.632653061	0.077764695	589835, 585934, 589392, 577928	55	4	183	3.327272727	0.999998655	1	1
	Biological Process	GO:0007017~microtubule-based process	3	4.109589041	0.06469339	373241, 575406, 591043	10	8	162	6.075	0.999919737	1	1
	Cellular Component	GO:0005875~microtubule associated complex	3	4.109589041	0.011451821	373241, 575406, 591043	13	3	183	14.07692308	0.339424825	0.412265572	0.412265572
	Cellular Component	GO:0016021~integral component of membrane	7	9.589041096	0.060588203	592207, 579307, 579243, 588706, 585029, 105438010, 594397	13	48	183	2.052884615	0.89460589	0.496871214	0.496871214
4	Cellular Component	GO:0031224~intrinsic component of membrane	7	9.589041096	0.066621097	592207, 579307, 579243, 588706, 585029, 105438010, 594397	13	49	183	2.010989011	0.916422902	0.496871214	0.496871214
	Cellular Component	GO:0044430~cytoskeletal part	3	4.109589041	0.069009891	373241, 575406, 591043	13	7	183	6.032967033	0.923788166	0.496871214	0.496871214
	Cellular Component	GO:0015630~microtubule cytoskeleton	3	4.109589041	0.069009891	373241, 575406, 591043	13	7	183	6.032967033	0.923788166	0.496871214	0.496871214
	Molecular Function	GO:0003824~catalytic activity	11	15.06849315	0.051247748	579307, 578700, 587263, 586173, 373241, 586702, 585029, 105438010, 590365, 594397, 574551	15	86	183	1.560465116	0.987954167	1	1
	Molecular Function	GO:0016798~hydrolase activity, acting on glycosyl bonds	3	4.109589041	0.091617021	587263, 586702, 105438010	15	7	183	5.228571429	0.999687683	1	1
5	Biological Process	GO:0044763~single-organism cellular process	5	19.23076923	0.046405712	575157, 580863, 373276, 591240, 593362	5	76	162	2.131578947	0.990039682	1	1
	Biological Process	GO:0044699~single-organism process	5	19.23076923	0.060178132	575157, 580863, 373276, 591240, 593362	5	81	162	2	0.997571053	1	1
